# neoDL: a novel neoantigen intrinsic feature-based deep learning model identifies IDH wild-type glioblastomas with the longest survival

**DOI:** 10.1186/s12859-021-04301-6

**Published:** 2021-07-23

**Authors:** Ting Sun, Yufei He, Wendong Li, Guang Liu, Lin Li, Lu Wang, Zixuan Xiao, Xiaohan Han, Hao Wen, Yong Liu, Yifan Chen, Haoyu Wang, Jing Li, Yubo Fan, Wei Zhang, Jing Zhang

**Affiliations:** 1 Key Laboratory for Biomechanics and Mechanobiology of Ministry of Education, Beijing Advanced Innovation Centre for Biomedical Engineering, School of Engineering Medicine, School of Biological Science and Medical Engineering, Beihang University, No.37 Xueyuan Road, Haidian District, Beijing, 100083 People’s Republic of China; 2 Department of Molecular Neuropathology, Beijing Neurosurgical Institute, Capital Medical University, Beijing, 100070 People’s Republic of China; 3 Department of Neurosurgery, Beijing Tiantan Hospital, Capital Medical University, No. 119 South Fourth Ring Road West, Fengtai District, Beijing, 100070 People’s Republic of China

**Keywords:** IDH wild-type glioblastoma, Peptide-features, Prognosis, Deep learning, Immunology

## Abstract

**Background:**

Neoantigen based personalized immune therapies achieve promising results in melanoma and lung cancer, but few neoantigen based models perform well in IDH wild-type GBM, and the association between neoantigen intrinsic features and prognosis remain unclear in IDH wild-type GBM. We presented a novel neoantigen intrinsic feature-based deep learning model (neoDL) to stratify IDH wild-type GBMs into subgroups with different survivals.

**Results:**

We first derived intrinsic features for each neoantigen associated with survival, followed by applying neoDL in TCGA data cohort(AUC = 0.988, *p* value < 0.0001). Leave one out cross validation (LOOCV) in TCGA demonstrated that neoDL successfully classified IDH wild-type GBMs into different prognostic subgroups, which was further validated in an independent data cohort from Asian population. Long-term survival IDH wild-type GBMs identified by neoDL were found characterized by 12 protective neoantigen intrinsic features and enriched in development and cell cycle.

**Conclusions:**

The model can be therapeutically exploited to identify IDH wild-type GBM with good prognosis who will most likely benefit from neoantigen based personalized immunetherapy. Furthermore, the prognostic intrinsic features of the neoantigens inferred from this study can be used for identifying neoantigens with high potentials of immunogenicity.

**Supplementary Information:**

The online version contains supplementary material available at 10.1186/s12859-021-04301-6.

## Background

Glioblastoma is the most common aggressive primary brain tumor having profound genomic heterogeneity and high recurrence rate [1]. Although the survival of GBMs has improved with the advancement of modern combination therapies, the prognosis of most GBMs remains poor and varies considerably among patients [2], revealing a dismal median duration of 14 months [3, 4].

Neoantigens are from mutation-containing proteins that generate novel immunogenic epitopes [5]. High nonsynonymous mutation loads harbor more neoantigens presented to CD8+ T cells on restricted HLA-I subtypes [6–8], leading to stronger immunogenicity and better overall survival in melanoma [9], lung cancer [10], and colorectal tumors [11]. However, in gliomas, higher mutational load means increased tumor aggressiveness [12]. Neoantigens are pivotal in personalized immunetherapies, promoting tumor-specific T-cell responses and affecting antitumor immune responses in a number of preclinical models [13, 14]. Although high-quality neoantigen model performed well in identifying IDH wild-type GBMs with the longest survival [15], the number of high quality neoantigens were limited, making clinical application difficult. The occurrence and characterization of neoantigen in pan-cancer showed that all positions in neoepitopes containing more hydrophobic residues than the wild-type [16], but the comprehensive features of neoantigens associated with prognosis and immunoreaction in IDH wild-type GBM remain elusive.

Deep learning models can derive features from noisy and raw data by learning high-level representations [17, 18]. Their flexibility and adaptability lead to their wide application in biomedical imaging [19], showing excellent level-accuracy in precise diagnosis and prognostic stratification of colorectal [20], prostate [21, 22], melanoma [23], and gliomas [24]. Deep learning also demonstrates its strong abilities in predicting Glioma grades [25], Glioma genetic mutation [26] and survival [27]. Recently, neoantigen-based machine learning is reported to predict neoantigen immunogenicity in colon and lung adenocarcinomas [28].

Here, we present a neoantigen intrinsic feature based deep learning model (neoDL), successfully stratifying IDH wild-type GBMs of TCGA into different prognostic subgroups (Additional file 1: Figure S1). Our model was further validated in an independent data from Asian population, even demonstrating its strong predictive power in some higher-grade gliomas, including Classical, Classical-like, Glioblastoma, IDH wild-type, Mesenchymal-like. GBMs identified by neoDL with better prognosis enriched in development, and cell cycle. Our neoDL has important implications in diagnosis and prognosis of IDH wild-type GBMs, and helps identify GBMs who most likely benefit from neoantigen based personalized immunetherapy.

## Results and discussion

### Identification of neoantigen intrinsic features associated with the overall survival of IDH wild-type GBMs

**Fig. 1:**
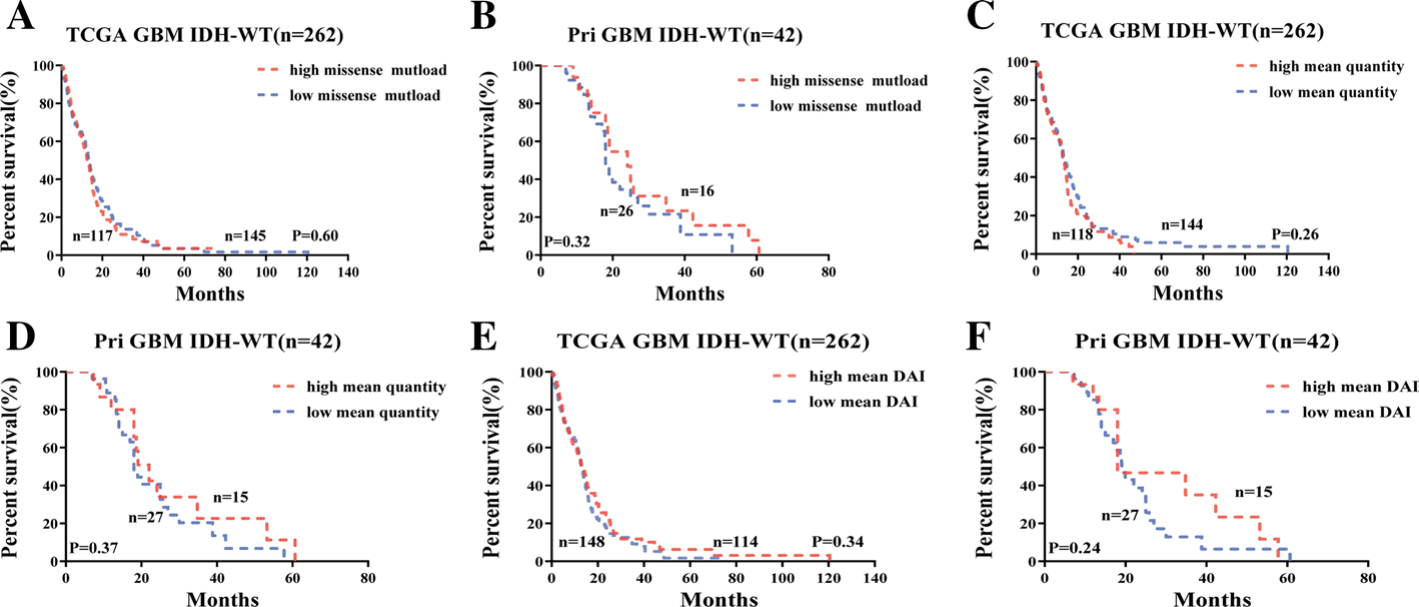
Missense mutational load, number of neoantigens and DAI fail to predict the survival of IDH wild-type GBMs. **A**–**F** Stratification of GBMs based on missense mutational load for **A** TCGA cohort, and **B** Pri cohort; on number of neoanigens for **C** TCGA cohort, and **D** Pri cohort; on DAI for **E** TCGA cohort, and **F** Pri cohort. n is number of patients. *p value* was from log-rank test. red (or blue) line is high (or low) mean value

Tumor mutational burden has been described as a predictor of tumor behavior and immunological response [29], with improved survival and immunotherapy response in melanomas [30], ovarian [31], and bladder carcinoma [32]. We calculated missense mutational load for 262 and 42 IDH wild-type GBMs in TCGA and Pri-cohort, respectively, finding no statistically significantly different overall survival between higher and lower mutation loads (Fig. 1A, B), consistent with the previous research [15]. Similarly, mutation loads were found either not prognostic or related to worse survival in 16 different glioma subtypes (Additional file 1: Figure S2). High missense mutational load harbored more neoantigens, rendering them more susceptible T-cell targets [33]. The neoantigen quantity also failed to predict the survival of IDH wild-type GBMs (Fig. 1C, D) and 16 different glioma subgroups (Additional file 1: Figure S3). DAI, defined as difference between binding affinity of wildtype and mutant-type peptides for MHC class I, was reported to be a better predictor of survival and immunogenicity in advanced lung cancer and melanoma [34]. We calculated the average DAI of each sample in both TCGA and Pri cohort, finding that DAI model failed in predicting the overall survival of IDH wild-type GBMs (Fig. 1E, F) and 16 different glioma subgroups (Additional file 1: Figure S4).

**Fig. 2:**
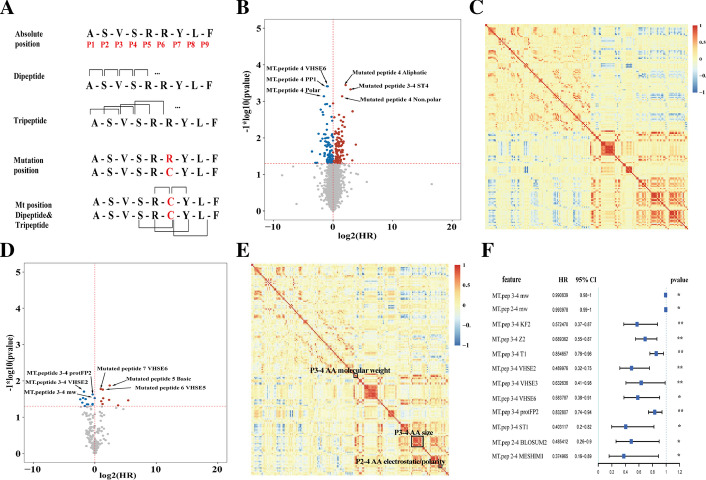
Prognostic neoantigen intrinsic features. **A** The classes of neoantigen-intrinsic features including characteristics at each absolute position, dipeptide, tripeptide, Mutant position, Mutant position dipeptide&tripeptide. Red numbers are the positions of amino acids in neoantigen. **B**, **D** Volcano plots representing log2(HR) (x-axis) and − log10 (*p value*) (y-axis) for each feature. **B** All features in TCGA cohort; **D** valid features in Pri cohort. Horizontal dashed line represents *p value* of 0.05 and the vertical one is HR of 1. Colored spot represents *p value* lower than 0.05, with red (or blue) representing HR above (or below) 1. **C**, **E** Correlations between valid features. **C** TCGA cohort; **E** Pri cohort. Red (or blue) is positive (or negative) correlation. **F** Forest plot for 12 peptide features in TCGA cohort.· *p value* < 0.1; *p value < 0.05; **p value < 0.01

An immunogenic neoantigen must possess structural and physical properties distinct enough to promote efficient recognition by T cells [35]. We calculated a total of 2928 features for each neoantigen and its wild-type peptide, including physical–chemical properties, AA (amino acid) features, and AA descriptors at each absolute position, composed-dipeptide and tripeptide at the site of mutation, and the dipeptides and tripeptides related to the mutation site, and complete sequence (Fig. 2A). The Shannon entropy and the AA composition were also calculated. We then performed Cox regression to estimate the association between the feature values and overall survival in IDH wild-type GBMs of TCGA, finding 189 prognostic features (termed as valid features) (Fig. 2B), among which the most significant positive associations were aliphatic AA in the absolute site 4 (Mutated peptide 4 Aliphatic), ST-scales4 descriptors of site 3 and 4 compose-dipeptide (Mutated peptide 3–4 ST4), and Nonpolar AA in the absolute site 4 (Mutated peptide 4 Non.polar). The most significant negative associations were theVHSE-scales6 descriptors, PP1 descriptors, and polar AA at the absolute position 4 (MT.peptide 4 VHSE6, MT.peptide 4 PP1, MT.peptide 4 polar). After calculating the correlation of valid features, we discovered that correlated feature modules were consistent across IDH wild-type GBM (Fig. 2C) and 16 different glioma subtypes in TCGA cohort (Additional file 1: Figure S5).

To further evaluate the prognostic value of valid features, we conducted Cox regression analysis in an independent data of Pri cohort, revealing 22 valid features significantly associated with the overall survival (Fig. 2D). The most significant positive associations were VHSE-scales6 at the 7 sites (Mutated peptide 7 VHSE6), basic AA at the site 5 (Mutated peptide 5 basic), VHSE-scales5 at the 6 site (Mutated pep 6 VHSE5). The most significant negative associations were mainly related to the characteristics of the positions 3 and 4 composed-dipeptide, including protFP2, VHSE-scales2, and molecular weight. Particularly, 12 features had shown strong mutual correlation, mainly associated with the molecular weight and molecular size/volume of the position 3,4 composed-dipeptide, and molecular electrostatic of the position 2–4 composed-tripeptide (Fig. 2E). Moreover, the 12 features were protective factors (HR < 1) in both TCGA cohort (Fig. 2F) and Pri cohort (Additional file 1: Figure S6).

### Deep learning model using neoantigen intrinsic features predicted IDH wild-type GBMs with better survival

**Fig. 3:**
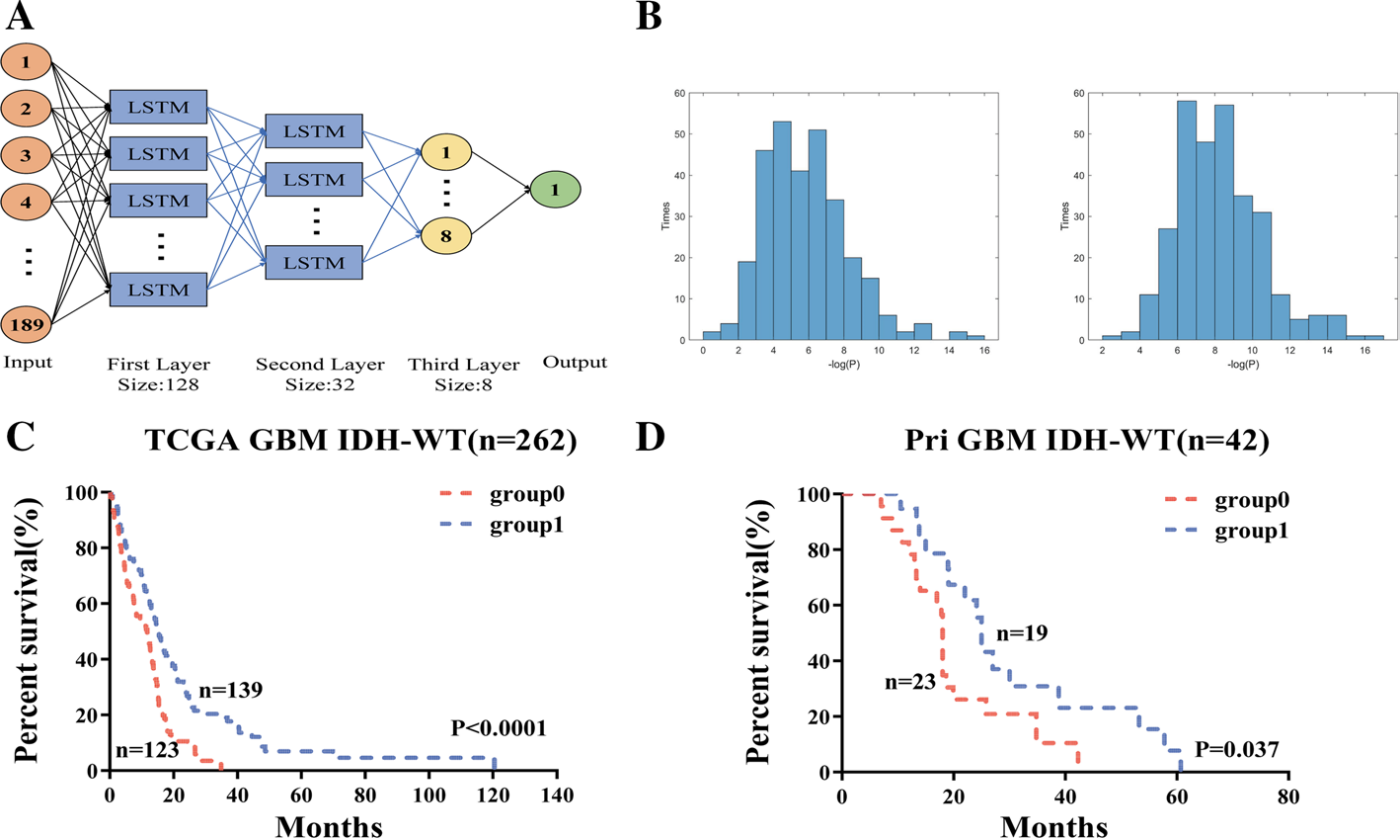
Deep learning model predicts survival of GBM. **A** Deep learning model diagram. **B** Left, *p value* distribution representing − log (*p value*) (x-axis) and times (y-axis) for 300 times in cross validation of TCGA. Right, Reliability verification of the trained model in 300 repeats with each randomly selecting 60% GBMs in TCGA. **C**, **D** Survival of GBMs stratified by the trained model in **C** TCGA cohort and **D** Pri cohort. Red line is the prediction label of 0, and blue line is 1. *p value* was from log-rank test. n is number of GBMs

Deep learning methods learn high-level representations with multilayer computational models, and are advantageous in learning high-dimensional datasets [17]. LSTM can avoid the problem of vanishing gradient [36], and has the ability to remember all previous data. To stratify IDH wild-type GBMs, we constructed a valid feature-based deep learning model including three hidden layers (two LSTM layers and one fully connected layer) with 128, 32, 8 nodes, respectively (Fig. 3A). We chose the Sigmoid function as neuron activation function for fully connected layer, MSE as the loss function and Adam as the iterative optimizer with the number of iterations set as 1000. When setting 1000 epochs when training the model, loss approaches to zero and accuracy approached to 100%. Predicting accuracy in cross validation continuously remained at a high level (over 90%), showing that the model was not over-fitting (Additional file 1: Figure S7). The samples in TCGA cohort (containing 262 labeled samples) were used as training data, while the samples in Pri cohort (containing 42 unlabeled samples) as external testing data. TCGA cohort was labeled based on the result of hierarchical k-means clustering, which stratified the data into a short-term survival group (cluster = 1, n = 126) and a long-term survival group (cluster = 2, n = 136).

To validate the reliability of the deep learning model, we performed 300 random trials with each splitting the samples into training set and testing set at the ratio of six vs four. The two sets were extracted separately from short- and long-term survival group with the specific ratio, thus the training set contains 60% of cluster 1 samples (n = 76) and 60% cluster 2 samples (n = 82). In each trial, the parameters learned in the training set were applied in the testing set. In 275 out of 300 trials, IDH wild-type GBMs in TCGA were successfully separated into two significantly different prognostic subgroups (*p* value < 0.05) (Fig. 3B left). The optimal parameter settings were determined and applied to randomly selected 60% of IDH wild-type GBMs in TCGA. In 299 of 300 randomly selected 60% of IDH wild-type GBMs in TCGA, our trained deep learning model successfully separated patients into two subgroups with significantly different overall survival (Fig. 3B right), demonstrating the stability and reliability of our model. We then applied the trained model to stratify all IDH wild-type GBMs in TCGA into two prognostic subgroups (AUC = 0.988, *p* value < 0.0001, Fig. 3C, Additional file 1: Table S7). As an independent validation, we successfully applied the trained model to separate IDH wild-type GBMs in an independent data (Pri GBM cohort) into two prognostic subgroups (*p* value = 0.037, Fig. 3D). We also successfully applied the trained model to divide patients into two different prognostic subgroups for GBM, IDH wildtype, Classical, Classical-like, Mesenchymal-like subtypes in TCGA pan-glioma cohort (*p* value < 0.05 for all subtypes) (Additional file 1: Figure S8). The flow chart of the neoDL model was visualized (Additional file 1: Figure S1).

### The prognostic characteristics of 12 protective intrinsic features

**Fig. 4:**
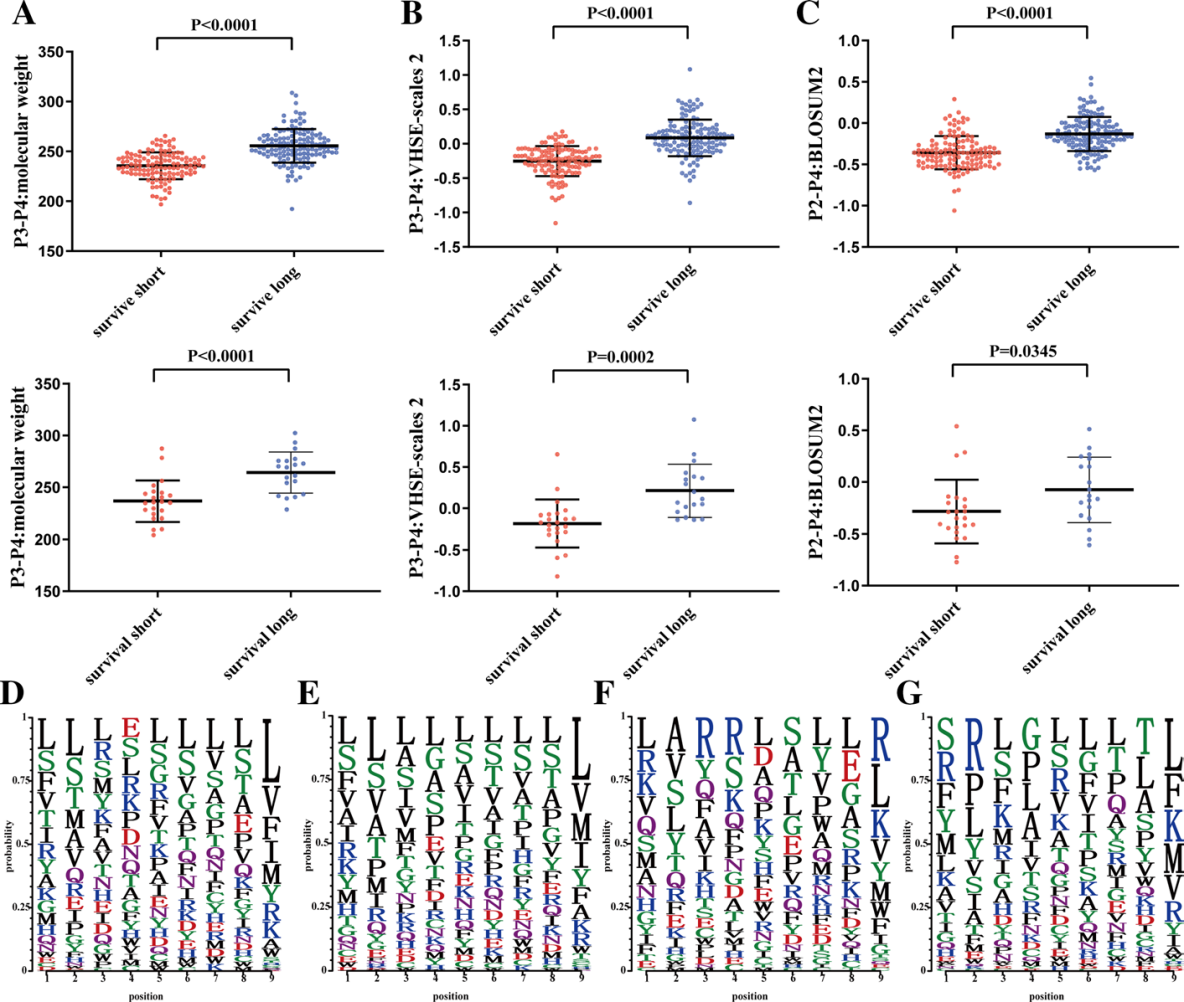
Characteristics of prognostic features. **A**–**C** Comparison of feature values between long- and short-term survival IDH wild-type GBMs. **A** molecular weight of dipeptide composed with sites 3 and 4. **B** VHSE-scales2 of dipeptide composed with sites 3 and 4. **C** BLOSUM2 of tripeptide composed with sites 2 and 4. The upper and the lower panels are TCGA cohort and Pri cohort, respectively. *p value* was from unpaired T test. **D**–**G** Comparison of the amino acid occurrence frequency for each position between the two groups. **D** Long- and **E** short-term survival patients in TCGA cohort. **F** Long- and **G** short-term survival patients in Pri cohort. The letter size is proportional with the occurrence

To characterize the 12 protective intrinsic features in the molecular weight, molecular size of dipeptide, and molecular electrostatic potential of tripeptide, we compared their distributions in the short- and long-term survival IDH wild-type GBMs. Compared with the short-term survival GBMs, the long-term survival patients exhibited statistically significantly higher molecular weight of dipeptide at the site 3 and 4 (*p value* < 0.05; Fig. 4A; Additional file 1: Figure S9a), molecular size-related features (Kidera Factors 2, Z-scale 2, T-scale 1, protFP2, VHSE-sclae 2, VHSE-sclae 3, VHSE-sclae 6, ST-scale 1) (*p value* < 0.05; Fig. 4B; Additional file 1: Figure S9b) and the electrostatic potential related features (BLOSUM2 and MESHIM1) (*p value* < 0.05; Fig. 4C; Additional file 1: Figure S9c) in both TCGA and Pri-cohort.

Univariate and multi-variate Cox regression [37] analysis demonstrated that two of 12 features (VHSE2 and protFP2) were associated with the overall survival in the two cohorts (Additional file 1: Table S1, Additional file 1: Table S2, Additional file 1: Table S3 and Additional file 1: Table S4). Kaplan Meier analysis demonstrated statistically significantly different overall survival between the low-value (below mean) and high-value (above mean) groups of IDH wild-type GBMs stratified by the two features. The patients with high-value (above mean) had a significantly longer overall survival (for protFP2: *p value* = 0.002 in TCGA cohort and *p value* = 0.03 in Pri cohort; for VHSE2: *p value* = 0.018 in TCGA cohort and *p value* = 0.11 in Pri cohort) (Additional file 1: Figure S10a–b). Furthermore, the two feature-based stratification of the IDH wild-type GBMs were found independent of age and mutational load. The two features also exhibited strong correlations (R = 0.87, *p value* < 2.2e−16 for TCGA; R = 0.91, *p value* < 2.2e−16 for Pri Cohort) (Additional file 1: Figure S10c).

The distributions of amino acid residue for neoantigens between long- and short-term survival groups were examined, revealing that the ratios of amino acid residues at positions 3 and 4 were significantly different (Fig. 4D–G). At the site 3, the patients with neoantigens containing a lower frequency of L and S amino acids and a higher frequency of R amino acid survived longer than those with the opposite frequencies in both cohorts. The enrichment of residues R and S at site 4 of neoantigens were evident in the long-term survival of IDH wild-type GBMs. The ratios of L and G at site 4 of neoantigens increased in the short-term survival patients.

### Tumor purity and functional annotation of gene expression in GBM

**Fig. 5:**
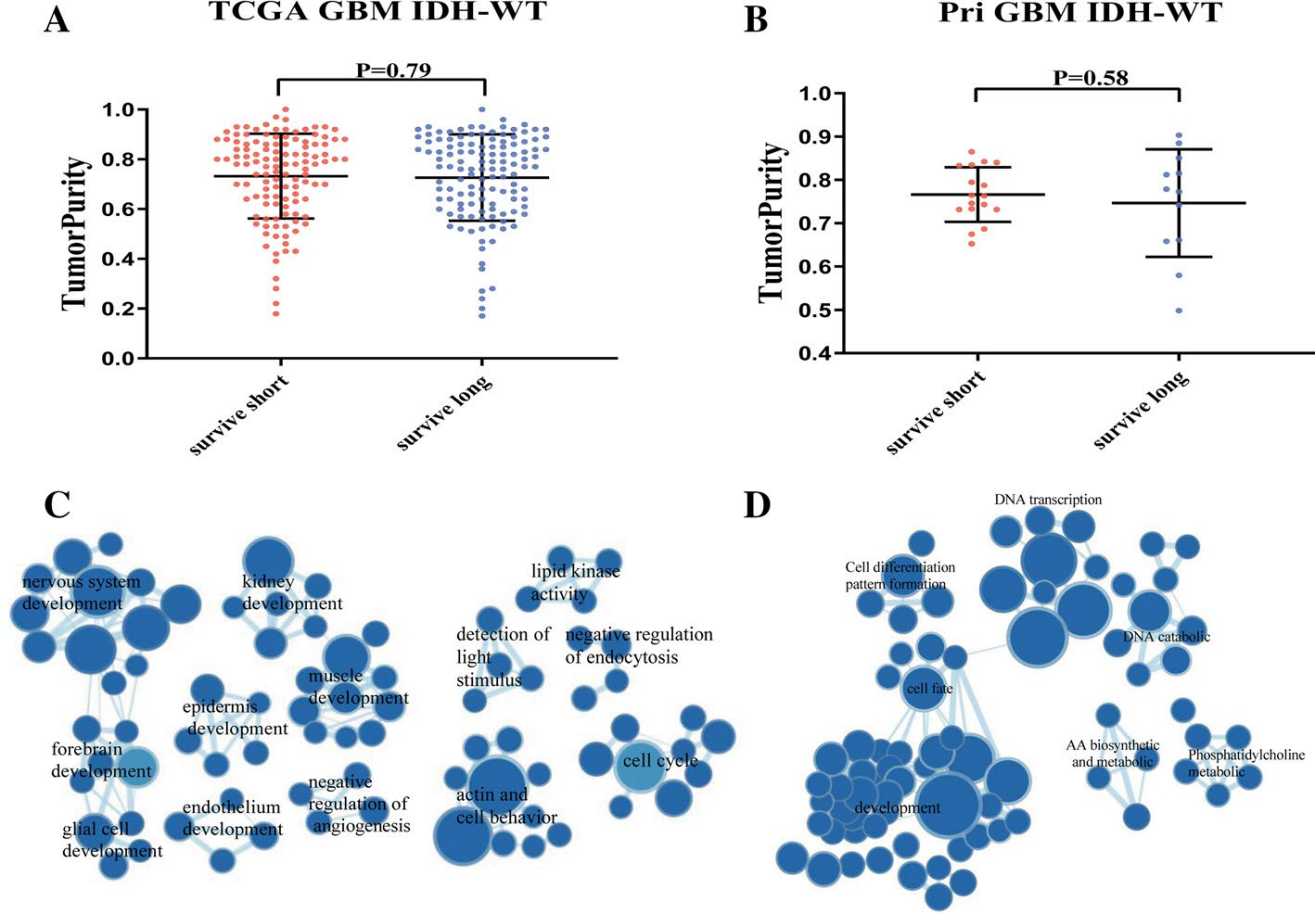
Tumor Purity and enriched gene network in GBM. **A**, **B** Tumo purity between long- and short-term survival groups. **A** TCGA cohort. **B** Pri cohort. *p value* was from two-tailed student T test. **C**, **D** Enriched gene network in TCGA cohort (**C**) and Pri cohort (**D**). Nodes represent GO terms with size proportional to number of genes. Lines are the fraction of genes shared between groups

We calculated the tumor purity, immune score, and stromal score using gene expression data for each patient in both TCGA and Pri cohorts. No significant differences were observed between long- and short-term survival of IDH wild-type GBMs (Fig. 5A, B for tumor purity, Additional file 1: Figure S11a for immune scores and S11b for stromal scores). No correlations were discovered between purity levels and mutational burden (Additional file 1: Figure S11C).

To understand the mechanisms in transcriptomic architecture, we conducted GSEA [38, 39], an algorithm for determining whether a set of genes differs between two biological states, between long- and short-term survival groups of IDH wild-type GBMs in both TCGA and Pri cohorts, respectively. Enrichment map analysis of deregulated GO terms in TCGA data demonstrated that GO terms related to development and cell cycle were enriched in long-term survival patients (Fig. 5C, Additional file 1: Table S5 and Additional file 1: Table S6). In Pri cohort, the most significant biological processes enriched in longer-survived GBMs were development associated GO terms such as epidermis development, cell cycle, which were also identified in TCGA cohort (Fig. 5D).

## Conclusion

In this paper, we presented a prognostic prediction deep learning model based on neoantigen intrinsic features. Although several survival prediction models have been reported based on the expression of several genes [40–42] or medical images [43, 44], they are not related to neoantigens and immune response. As neoantigens are associated with tumor-specific T-cell responses and anti-tumor immune responses, the method we provided can help predict the prognosis of IDH wild-type GBM patients who will likely benefit from neoantigen based personalized immunetherapy.

Our model achieved good predictive performances in two independent data cohorts of IDH wild-type GBMs (KM: log rank *p* value < 0.0001 in TCGA cohort; 0.037 in Pri cohort) and even in some other high-grade glioma subtypes. Currently, the vast majority of deep learning models (such as DeepLearningModel [45] and PASNet [46]) are based on gene expression, clinical information and medical image data for learning modeling, and there are few predictions of GBM patient survival based on the nature of neoantigens. We compared our neoDL with them and found that neoDL performed better than DeepLearningModel and PASNet (Additional file 1: Table S7). GBMs predicted by our model to have better survival enriched in development and cell cycle. Two correlated neoantigen features (VHSE2 and protFP2) were identified to stratify GBMs into a high- and low-value subgroup with significant different survival independent of other clinical and pathological characteristics.

Of 189 valid features, 12 protective features associated with survival in both cohorts were amino acid molecular weight, molecular size/volume, and electrostatic potential/polarity, which were characterized by close relation with the amino acid properties at the positions 3 and 4 of the neoantigen, confirmed by the amino acid distributions between different survival groups. The features at the site 3 and 4 of the neoantigen may have potential effects on the survival of GBMs and immunotherapy response, and they are worthy of further investigation.

In this study, we focused on sequence structure in this study, but not on secondary and tertiary protein structure. More features may be integrated into the model to improve predictive power, which shall be resolved in the future. The deep learning methods (such as DeepCoxPH [47] and FuzzyDeepCoxPH [48]) reported to be effective in other scenario can also be used to augment the prognostic evaluation and improve decision-making in glioma. To predict the patients’ outcome, more studies related to generalizability test are still in need.

## Methods

### Data description

Mutations and clinical information were from the ATLAS-TCGA pan-glioma study [49]. Gene expression data (G4502A) at level 3 were from TCGA Data portal. We termed the data from TCGA as TCGA cohort. Mutations, RNAseq data, and clinical information in Asian population were from a recently published cohort [50], designated as Pri cohort. The samples that not diagnosed as IDH wild-type GBM or have clinical information lost were removed, resulting in 268 and 46 samples in the two cohorts, respectively.

A neoepitope with strong affinity for MHC (${IC}_{50}$ $\le$ 500 nM) may be a more robust neoantigen candidate if the paired wild-type epitope has a poor affinity for MHC (${IC}_{50}$ > 500 nM) [51]. The neoantigens and their corresponding wild-type peptides for each sample in TCGA cohort and Pri cohort were from our previous study [15], which used missense mutations to generate all possible 9-mer peptides and defined the mutant 9-mer peptides as neoantigens when the ${IC}_{50}$ of mutant-type peptides was < 500 nM and the corresponding wild-type binder > 500 nM.

### Feature calculation for neoantigens

262 (TCGA cohort) and 42 samples (Pri cohort) with detected neoantigens remained in the downstream analysis. 2928 features (Additional file 1: Table S8) were extracted from 2263 neoantigens (2081for TCGA cohort; 182 for Pri cohort) using R: ‘Peptides’(v2.4.2) for 66 amino acid descriptors and 10 physical–chemical properties, aaComp for amino acid composition of neoantigens, and custom scripts for features from Shannon entropy (Additional file 1).

### Prognostic feature selection

The features were calculated for all neoantigens and wild-type peptides, followed by averaging all feature values in each patient. Univariate Cox regression analysis was to predict the prognostic impact of each feature. 189 features with *p value* ≤ 0.05 were termed as valid features (Additional file 1: Table S9). Correlation matrix of the valid features were visualized through heatmaps using R:‘pheatmap’.

### Hierarchical k-means clustering

Hierarchical k-means clustering was applied upon Z-Score-transformed valid features to stratify patients into two clusters using the "hkmeans" command of the R: ‘factoextra’ (version 1.0.7).

### Deep-learning model construction

The valid features in TCGA cohort were used to train deep learning model. The groups from hierarchical k-means clustering were used as labels. Z-Score-transformed were applied upon feature values of valid features to avoid gradient disappearance problem. The LSTM (Long short-term memory) deep learning model was built with three hidden layers (two LSTM layers and one fully connected layer), with each containing 128, 32, and 8 nodes, respectively. We chose the Sigmoid function as neuron activation function for fully connected layer, since we wanted to map the original statistics to a single number with domain of 0–1 through learning, which refered to the final classification result. The original data were normalized using z-score, therefore no serious gradient vanishing problem would be caused when using Sigmoid fuction as activation function. For hyperparameters, we chose MSE as the loss function and Adam as the iterative optimizer with the number of iterations set as 1000. MSE is a commonly used loss function in regression problem, thus we utilized such function to calculate the preference of a sample. The initial connection weights and biases of each layer were randomly generated, and end up reaching stable parameters through training iterations.

### Leave one out cross validation (LOOCV)

Cross validation was performed as follows. TCGA cohort was randomly separated into training and test sets at the ratio of six to four. To obtain the optimal model, the above randomizations were conducted 300 times. For each randomization trial, the model parameters were trained in the training sets. The trained model was applied to stratify the test set into two subgroups, followed by Kaplan–Meier survival analysis. *p value* ≤ 0.05 were regarded as statistically significant. The optimal parameter settings were determined from 300 randomization trials. To evaluate the reliability, the trained model were then applied to randomly selected 60% of IDH wild-type GBMs in TCGA, which were repeated 300 times.

### Independent validation

Pri cohort was used as an external test data to test the performance of the trained model, which divided patients into long- and short-term survival clusters. Other glioma subtypes from TCGA were also used to test the trained model, including Astrocytoma, Classical-like, Classical, Codel, Glioblastoma, G-CIMP-high, IDH-MT-codel, IDH-MT-noncodel, IDH-MT, IDH-WT, Mesenchymal-like, Mesenchymal, Neural, Oligodendroglioma, Proneural and OligoAstrocytoma.

### Tumor purity estimation

Tumor purities were estimated by ESTIMATE [52] using R: ‘estimate’(version 1.6.7). There were 242 (TCGA cohort) and 29 IDH wild-type GBMs (Pri cohort) with gene expression profiles available.

### GO enrichment analysis

GO enrichment analysis was conducted using Gene Set Enrichment Analysis (GSEA 4.0.3). The GO terms were from the Molecular Signatures Database (c5.all.v6.2.symbols.gmt). Gene sets with FDR < 0.05 were considered as differentially expressed, and visualized using Cytoscape [53]. The GSEA results were shown in Additional file 1: Table S5 and Additional file 1: Table S6.

### Statistical analysis

Variables between groups were compared by the unpaired T test, a Parametric test method which compares two different subjects. Correlations were evaluated by Pearson correlations. Kaplan–Meier survival and Cox regression analyses were performed using R: survminer" and "survival". *p value* ≤ 0.05 was determined as significance in all tests. All analyses were conducted in R and Python.

## Supplementary Information

**Additional file 1.** Description of neoDL and supplementary results.

## Data Availability

All data are from original researches properly cited in Material and methods. neoDL and the intrinsic features of neoantigens calculated for both TCGA cohort and Pri cohort are at github (https://github.com/zhangjbig/neoDL).
