# Supplementary material for: neoDL: a novel neoantigen intrinsic feature-based deep learning model identifies IDH wild-type glioblastomas with the longest survival
Source: BMC Bioinformatics. 2021 Jul 23;22:382. doi: 10.1186/s12859-021-04301-6 (PMC8299600; doi:10.1186/s12859-021-04301-6)
Supplement: Supplementary file 1 — Additional file 1. Description of neoDL and supplementary results. [file 12859_2021_4301_MOESM1_ESM.pdf]

neoDL: A novel neoantigen intrinsic feature-based deep learning modelidentifies IDH wild-type glioblastomas with the longest survival

Ting Sun[1,*], Yufei He[1,*], Wendong Li[1,*], Guang Liu[1], Lin Li[1], Lu Wang[1], Zixuan Xiao[1], Xiaohan Han[1], Hao Wen[1], Yong Liu[1], Yifan Chen[1], Haoyu Wang[1], Jing Li[1], Yubo Fan[1,#], Wei Zhang[2,3,#], Jing Zhang[1,#]

Materials and Methods

Supplementary Figure S1. Flow chart of neoDL.

Supplementary Figure S2. Survival of glioma patients stratified according to missense mutational load.

Supplementary Figure S3. Survival of glioma patients stratified according to absolute number of neoantigens.

Supplementary Figure S4. Survival of glioma patients stratified according to differential agretopicity index (DAI).

Supplementary Figure S5. Heat map representing Spearman correlation between each valid feature.

Supplementary Figure S6. Forest plot for 12 peptide features in Pri cohort.

Supplementary Figure S7. Relationship between the number of iterations and loss/accuracy.

Supplementary Figure S8. Survival of TCGA glioma patients stratified by deep learning model.

Supplementary Figure S9. Comparison of the similarity of valid feature values between long-term survival and short-term survival groups of IDH wild-type GBM in two cohorts.

Supplementary Figure S10. Survival of glioma patients stratified according to 2 feature values， and analysis of the correlations between these features in two cohorts.

Supplementary Figure S11. Comparison of the similarity of immune score and stromal scorebetween two groups and the correlation analysis between purity and mutation load.

Supplementary Table S1. Multivariate Cox regression analysis including position 3-4 composed-dipeptide VHSE-scale 2 value, mutation load and age for TCGA IDH wild type GBM (n=262).

Supplementary Table S2. Multivariate Cox regression analysis including position 3-4 composed-dipeptide VHSE-scale 2 value, mutation load and age for Pri IDH wild type GBM (n=42).

Supplementary Table S3. Multivariate Cox regression analysis including position 3-4 composed-dipeptide protFP 2 value, mutation load and age for TCGA IDH wild type GBM (n=262).

Supplementary Table S4. Multivariate Cox regression analysis including position 3-4 composed-dipeptide protFP 2 value, mutation load and age for Pri IDH wild type GBM (n=42).

Supplementary Table S5. Functional annotation for the lists of genes differentially expressedanalyzed by GSEA in TCGAcohort.

Supplementary Table S6. Functional annotation for the lists of genes differentially expressedanalyzed

by GSEA in Pri cohort.

Supplementary Table S7. Comparison of the effects of neoDL with two other methods.

Supplementary Table S8. Total features uesd in neoDL.

Supplementary Table S9. 189 features characterized by immunogenicity after screening.

Materials and Methods

Data Description

Mutations and clinical information were downloaded from the ATLAS-TCGA pan-glioma study(Ceccarelli, et al., 2016). Gene expression microarray data with Agilent chip (G4502A) at level 3 were downloaded from TCGA Data portal. We termed the data from TCGA as TCGA cohort. Mutations, RNAseq gene expression data, and clinical information in Asian population were collected from a recently published cohort(Wang, et al., 2016), designated as Pri cohort. The samples that were not diagnosed as IDH wild-type GBM or did not have clinical information were removed, resulting in 268 and 46 samples in the two cohorts, respectively.

A neoepitope with strong affinity for MHC ($IC50$ equal or less than 500 nM) may be a more robust neoantigen candidate if the paired wild-type epitope has a poor affinity for MHC ($IC50$ greater than 500 nM)(Wood, et al., 2018). The neoantigens for each sample in both TCGA cohort and Pri cohort were from our previous study(Zhang, et al., 2019), which used missense mutations to generate all possible 9-mer peptides and defined the mutant 9-mer peptides as neoantigens when the $IC50$ of mutant-type 9-mer peptides was less than 500 nM and the corresponding wild-type binder more than 500 nM. All the downstream analyses were based on the inferred neoantigens (the mutant peptides) and their corresponding wild-type peptides.

Feature calculation for neoantigens

For the purpose of extracting features from neoantigens, the samples with detected mutant

peptides remained in the downstream analysis, including 262 samples in the TCGA cohort and 42 samples in Pri cohort. A total of 2928 features were extracted from 2263 neoantigens (2081for TCGA cohort; 182 for Pri cohort) in the downstream analysis. Specifically, features used in the calculation were derived using the R package "Peptides"(v2.4.2) including 66 amino acid descriptors and physical-chemical properties (aliphatic, auto-correlation, auto-covariance, Boman index, theoretical net charge, cross-covariance, hydrophobic moment, hydrophobicity, instability, molecular weight). Additionally, the "aaComp" command was also used to describe amino acid features including Tiny, Small, Aliphatic, Aromatic, Non-polar, Polar, Basic, Acidic. Variables were derived by the presence (1) or absence (0) of each feature. Characteristic variables were performed in four conditions respectively, including the complete sequence, the site of mutation along with each antigen and the dipeptides/tripeptides related to the mutation site, each absolute position along each antigen and related dipeptide/tripeptide composition, and the difference of each feature in the mutated versus reference antigen.

The features, described overall content of a protein, for example, amino acid composition, were significant. Variables demonstrating presence(1) or absence(0) of each amino acid type following, including the first or last 3 amino acid residues or middle residues of each antigen, the first or last amino acid residues of each antigen, the first or last 2 amino acid residues or middle residues of each antigen.

To measure the complexity at the protein and residue level, we computed Shannon entropy of a protein and entropy of each type of residues using the following equations：

$$\mathrm{HS} = -\sum_{i=1}^{20} p_i log_2 \, p_i$$

$$HR_i = -p_i log_2 p_i$$

where HS is Shannon entropy of a protein sequence and HR_i is the entropy of a residue type i. p_i is the probability of the existence of a given amino acid in the sequence. We calculated the Shannon entropy of the mutant peptides and the difference of Shannon entropy in the mutant antigen versus reference antigen. Cancer is characterized by the accumulation of mutations, so the analysis of mutant positions is valid. Therefore, the Shannon entropy of the dipeptides/tripeptides related to the mutation site and the entropy difference of mutations process were performed. The entropy of a residue type was also calculated for the mutant peptides and reference peptides.

Prognostic feature selection

The features were calculated for all detected 9-mer mutated peptides and wild peptides. There were multiple types of mutations in each patient, resulting in a large number of mutant peptides in total. Thus, each feature value calculated in all the peptides detected in a patient was averaged as the final value. Univariate Cox regression analysis was performed here to predict the impact of each feature on prognosis. The threshold of P-value was set as 0.05, which means all the features with P-value lower than or equal to 0.05 were deemed as statistically significant (termed as valid features). A correlation matrix of the valid features was conducted, and visualized through heatmaps using the package 'pheatmap' in R language.

Hierarchical k-means clustering

Hierarchical k-means clustering was applied upon the Z-Score-transformed valid features to stratify patients into two clusters. Hierarchical k-means was performed using the "hkmeans" command of the R package 'factoextra' (version 1.0.7). The overall survival differences between

two clusters of patients were compared through Kaplan-Meier survival analysis.

Deep-learning model construction

The grouping results derived from hierarchical k-means clustering were used as labels, marking 0 and 1. The valid features in the TCGA cohort were used as training data to train the deep learning model. The input data were Z-Score-transformed valid features, in order to avoid gradient disappearance problem. The LSTM deep learning model was built with three hidden layers, including two LSTM layers and one fully connected layer, each layer containing 128, 32, and 8 nodes, respectively. We chose the Sigmoid function as neuron activation function for fully connected layer, since we want to map the original statistics to a single number with domain of 0-1 through learning, which refers to the final classification result. The original data are normalized using z-score, therefore no serious gradient vanishing problem would be caused when using Sigmoid fuction as activation function. For hyperparameters, we chose MSE as the loss function and Adam as the iterative optimizer with the number of iterations set as 1000. MSE is a commonly used loss function in regression problem, thus we utilized such function to calculate the preference of a sample. The maximum number of iterations was set as 1000. The initial connection weights and biases of each layer were randomly generated, and end up reaching stable parameters through training iterations.

Leave one out cross validation (LOOCV)

After determining the framework of the model, cross validation was a necessary step. Specifically, the training data was separated into two sections randomly with proportion of training and testing sets as 6 to 4. The training set was used to train the model to determine the unknown parameters, while the test set was used to validate the effect of the predicted parameters. To obtain the optimal model, the above process was carried out 300 times. Kaplan- Meier survival analysis was operated each time to see if the model can divide the samples into two groups with a statistically significant survival difference. Only groups with P-value lower than or equal to the threshold of 0.05 were regarded as statistically significant. Among 300 times trial, the more significant stratifications, the more stable our model is.

Independent validation

A model with fixed parameters corresponding to the lowest P-value was selected as the optimal model. To test the performance of the optimal model, Pri cohort was used as an external test data. The optimal model divided patients in the Pri cohort into long- and short-term survival clusters. Kaplan Meier analysis was conducted between the long- and short-term survival clusters in Pri cohort to test the predictive performance of the optimal model for IDH wild-type GBMs. Besides, other glioma subtypes from TCGA data were also tested by the model, including Astrocytoma, Classical-like, Classical, Codel, Glioblastoma, G-CIMP-high, IDH-MT-codel, IDH-MT-noncodel, IDH-MT, IDH-WT, Mesenchymal-like, Mesenchymal, Neural, Oligodendroglioma, Proneural and OligoAstrocytoma.

Tumor purity estimation

Tumor purities were estimated by ESTIMATE(Yoshihara, et al., 2013) from gene expression profiles. There were a total of 242 and 29 IDH wild-type GBMs in the TCGA cohort and Pri cohort with gene expression profiles available, respectively. The purity score was performed for each sample, using the R package 'estimate'(version 1.6.7). Meanwhile, the immune score and the stromal score were also estimated.

GO enrichment analysis

To identify differential genes expression between different groups, GO enrichment analysis was conducted using Gene Set Enrichment Analysis (GSEA 4.0.3)(Subramanian, et al., 2005), with 17814 and 23491 genes available in the TCGA cohort and Pri cohort, respectively. The GO terms were collected from the Molecular Signatures Database (c5.all.v6.2.symbols.gmt), including

cellular component, molecular function, and biological process. Number of Permutations was set to 1000 and gene set size filters were 15-500. Gene sets with FDR < 0.05 were considered as differentially expressed, and visualized using Cytoscape(Shannon, et al., 2003). The grouping results of GO terms were shown in Supplementary Table S5-S6.

Statistical Analysis

All statistical analyses were performed using R software, version 4.0.0. Continuous variables between groups were compared by the unpaired T test. Correlations between continuous variables were evaluated by Pearson correlation analyses. For all statistical analyses, the P value of 0.05 was taken as the significant threshold in all tests. Kaplan-Meier survival analysis curves were compared using a log-rank test and Multivariate survival analysis was performed by Cox regression model using R package "survminer" and "survival". All analyses were conducted in R language and Python language.

Ceccarelli, M., *et al.*(2016) Molecular Profiling Reveals Biologically Discrete Subsets and Pathways of Progression in Diffuse Glioma. *Cell*;164(3):550-563.

Shannon, P., *et al.*(2003) Cytoscape: a software environment for integrated models of biomolecular interaction networks. *Genome Res*;13(11):2498-2504.

Subramanian, A., *et al.*(2005) Gene set enrichment analysis: a knowledge-based approach for interpreting genome-wide expression profiles. *Proc Natl Acad Sci U S A*;102(43):15545-15550. Wang, J., *et al.*(2016) Clonal evolution of glioblastoma under therapy. *Nat Genet*;48(7):768-776. Wood, M.A., *et al.*(2018) Population-level distribution and putative immunogenicity of cancer neoepitopes. *BMC Cancer*;18(1):414.

Yoshihara, K., *et al.*(2013) Inferring tumour purity and stromal and immune cell admixture from expression data. *Nat Commun*;4:2612.

Zhang, J., *et al.*(2019) The combination of neoantigen quality and T lymphocyte infiltrates identifies glioblastomas with the longest survival. *Commun Biol*;2:135.

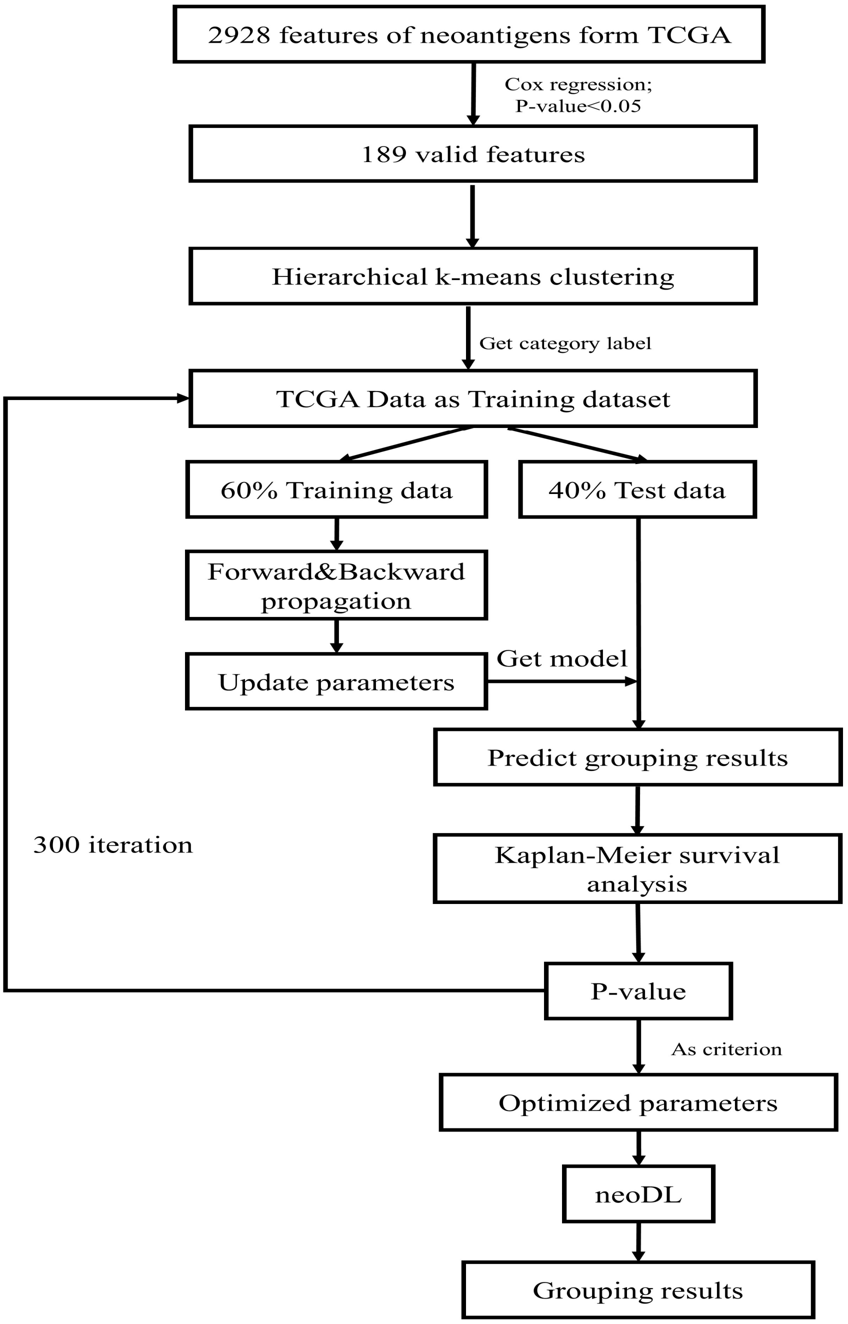

Supplementary Figure S1. Flow chart of neoDL.The flow chart contains three parts: extracting neoantigen-features, training process(that was done with 300 iterations) and applying in an independent data set.

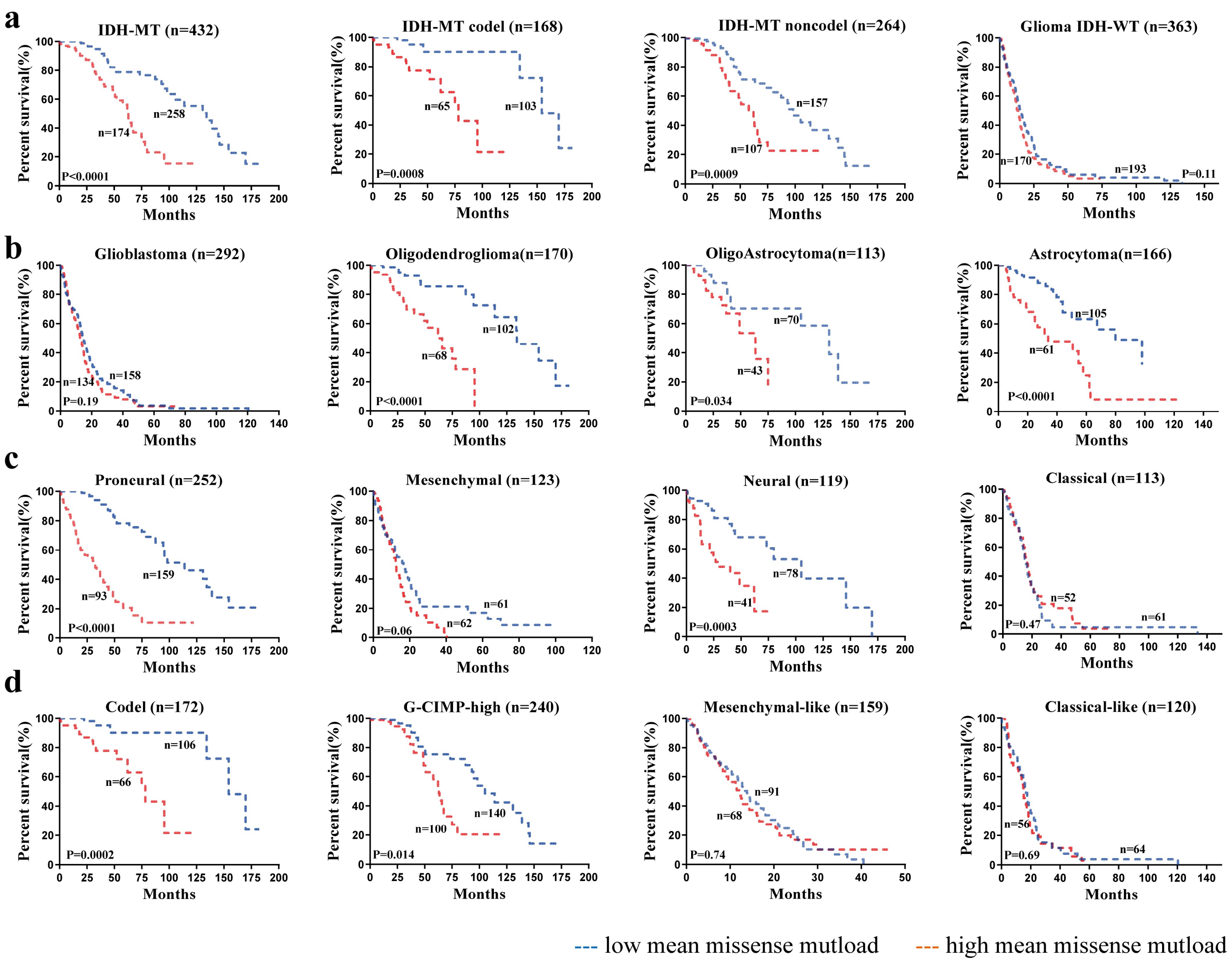

Supplementary Figure S2. Survival of glioma patients stratified according to missense mutational load. **a**, Glioma IDH status sub-groups; **b**, Glioma histology sub-groups; **c**, Glioma transcriptomic sub-groups; **d**,Glioma DNA methylation sub-groups. red line, high mean mutational load ; blue line, low mean mutational load; n, number of patients; p-value was determined using the log-rank test.

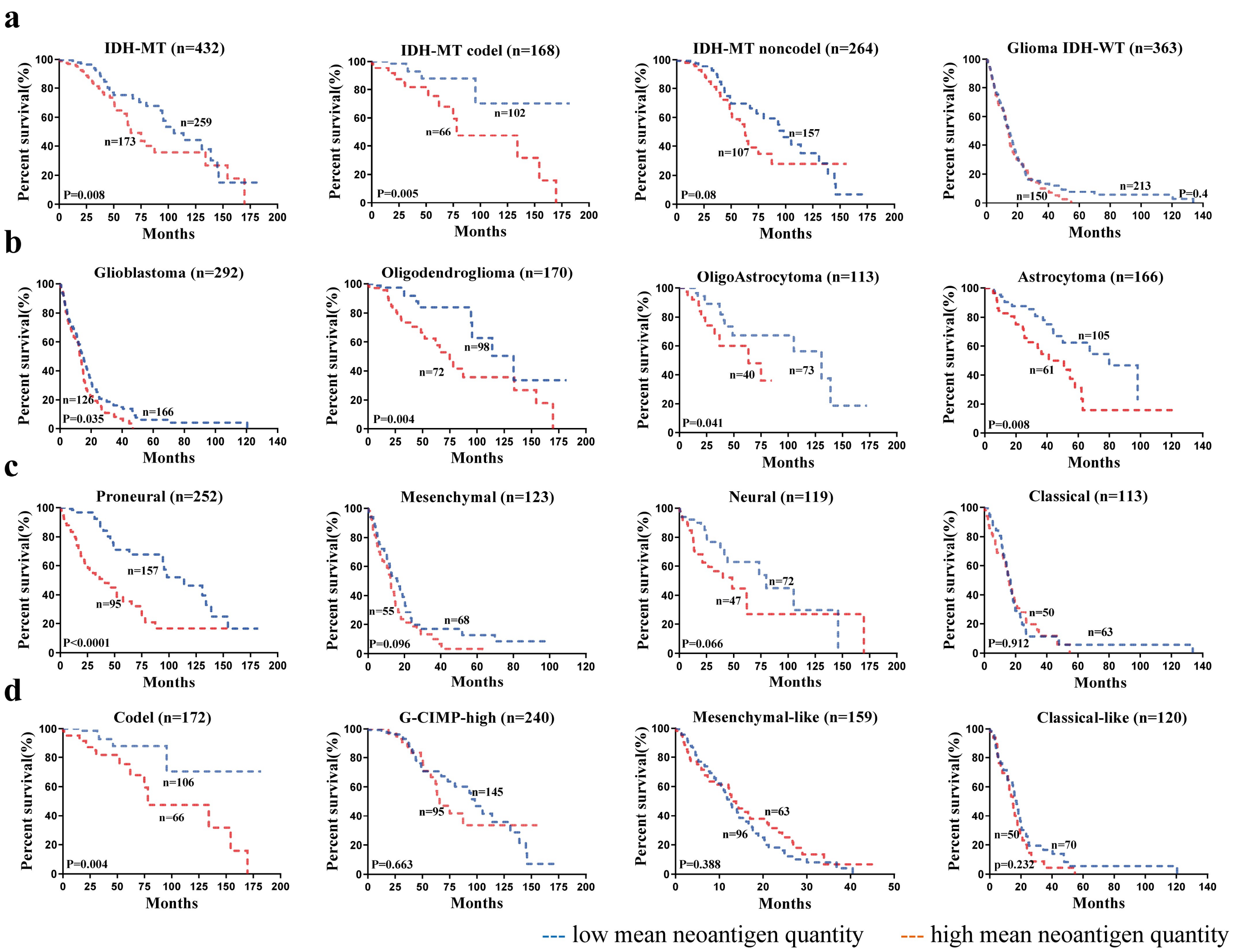

Supplementary Figure S3. Survival of glioma patients stratified according to absolute number of neoantigens. **a**, Glioma IDH status sub-groups; **b**, Glioma histology sub-groups; **c**, Glioma transcriptomic sub-groups; **d**,Glioma DNA methylation sub-groups. red line, high mean mutational load ; blue line, low mean mutational load; n, number of patients; p-value was determined using the log-rank test.

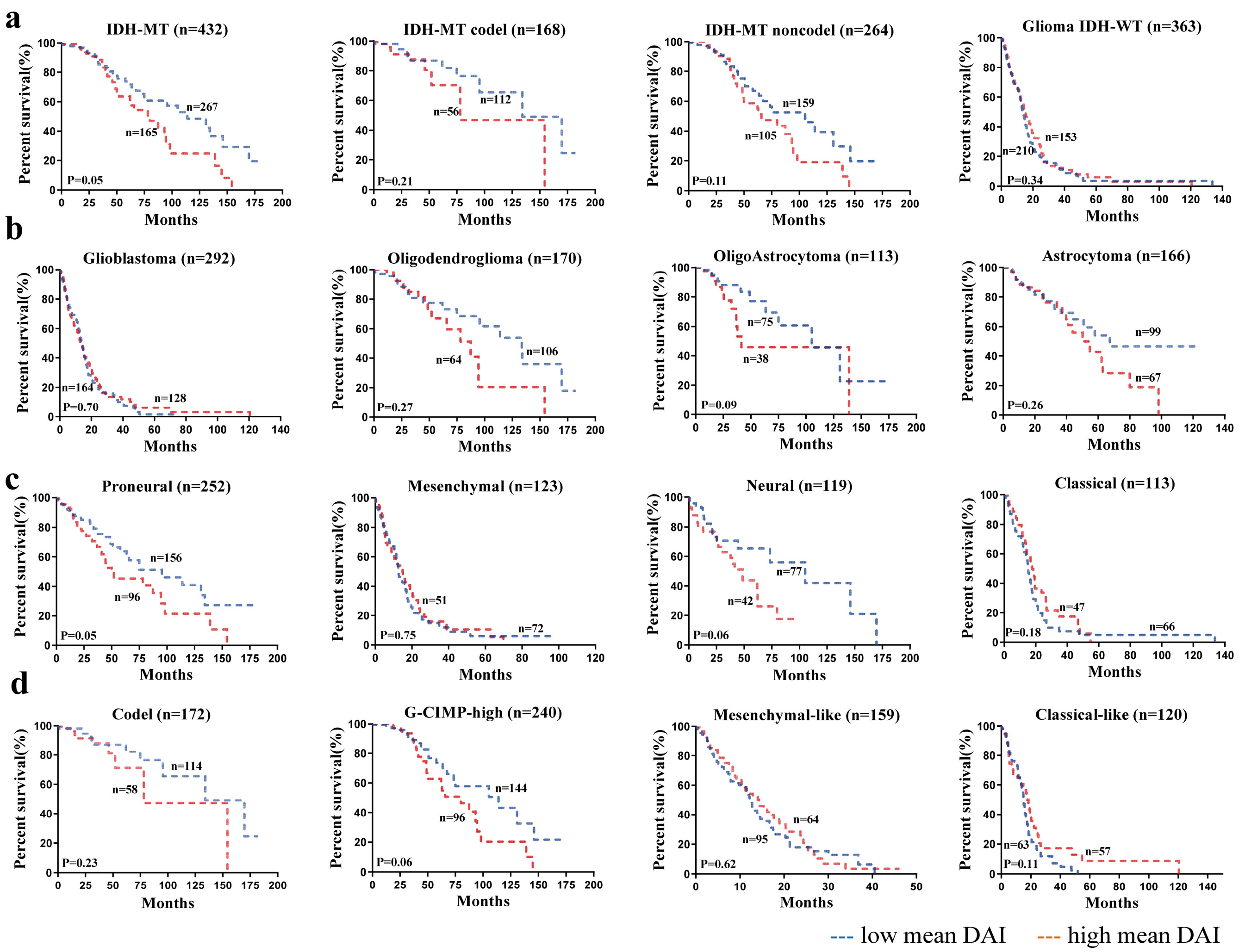

Supplementary Figure S4. Survival of glioma patients stratified according to differential agretopicity index (DAI). **a**, Glioma IDH status sub-groups; **b**, Glioma histology sub-groups; **c**, Glioma transcriptomic sub-groups; **d**,Glioma DNA methylation sub-groups. red line, high mean DAI; blue line, low mean DAI; n, number of patients; p-value was determined using the log-rank test.

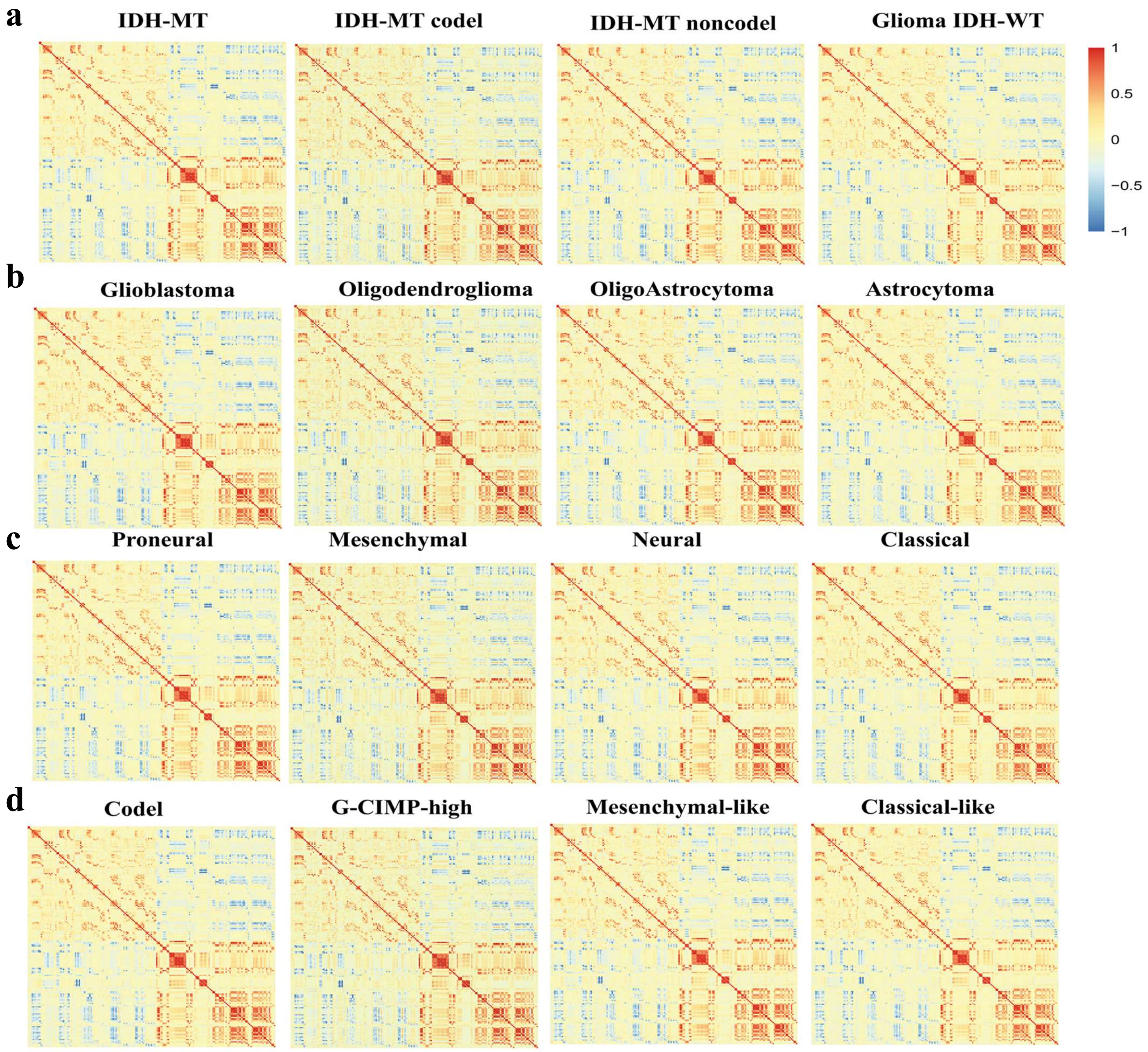

Supplementary Figure S5. Heat map representing Spearman correlations between each valid feature. Magnitude of the correlation coefficient represented by color. **a**, Glioma IDH status sub-groups; **b**, Glioma histology sub-groups; **c**, Glioma transcriptomic sub-groups; **d**,Glioma DNA methylation sub-groups.

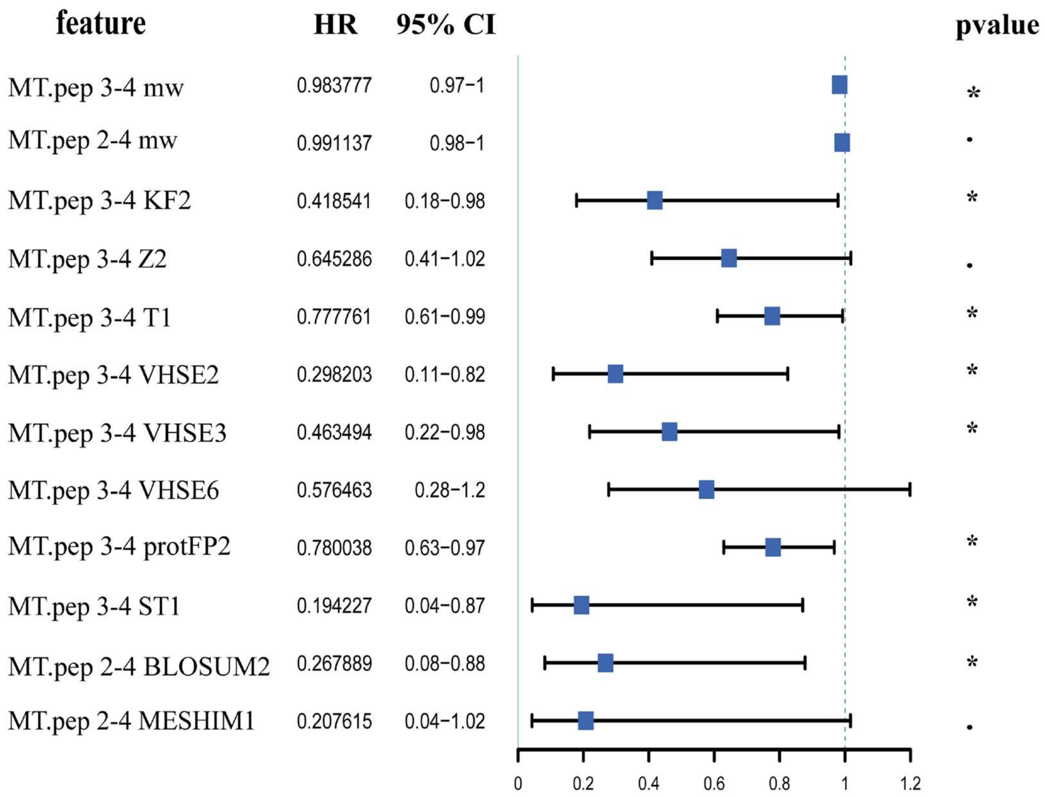

Supplementary Figure S6. forest plot for 12 peptide features in Pri cohort. · pvalue<0.1;* pvalue<0.05;** pvalue<0.01. HR value and pvalue derived by cox regression.

a

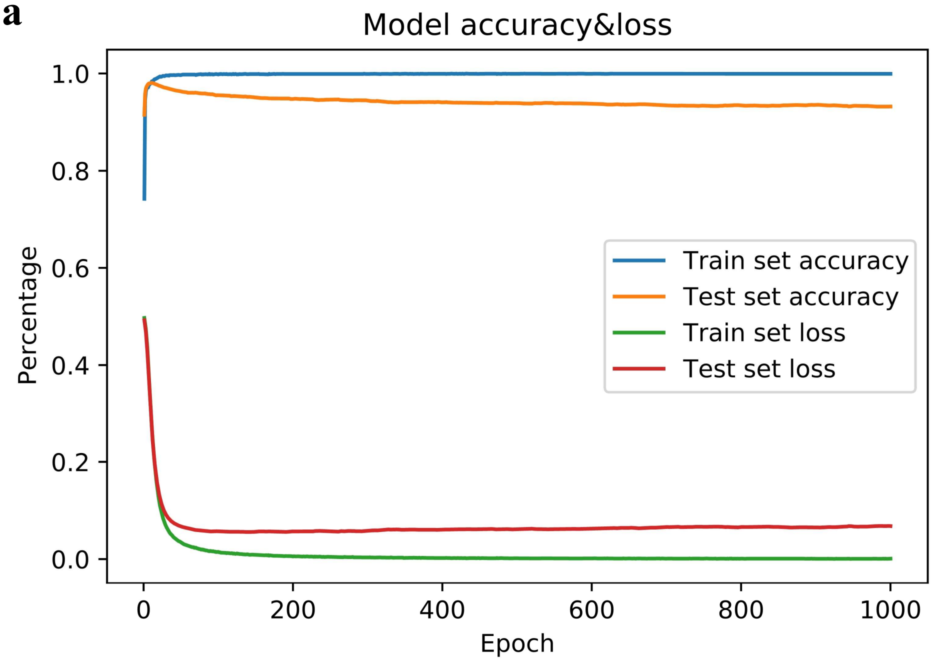

b

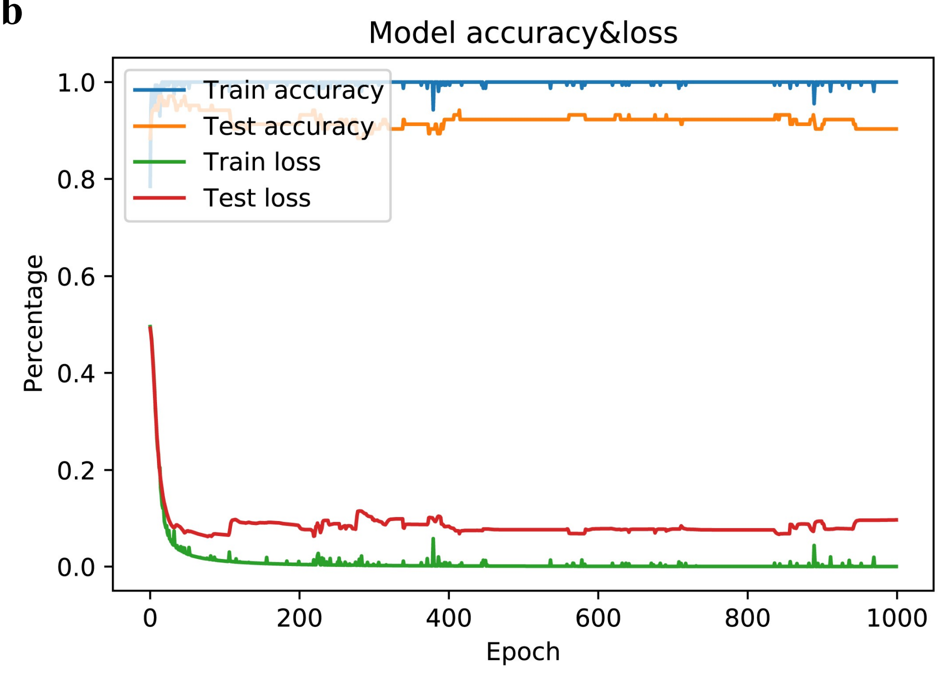

Supplementary Figure S7. a. Relationship between the number of iterations and loss/accuracy. The number of iterations is set to 1000, and the loss/accuracy change curves are the average value of 300 iteration. **b**. the process of gaining our optimized model. There are four lines shown below, which are train set accuracy, test set accuracy, train set loss, test set loss.

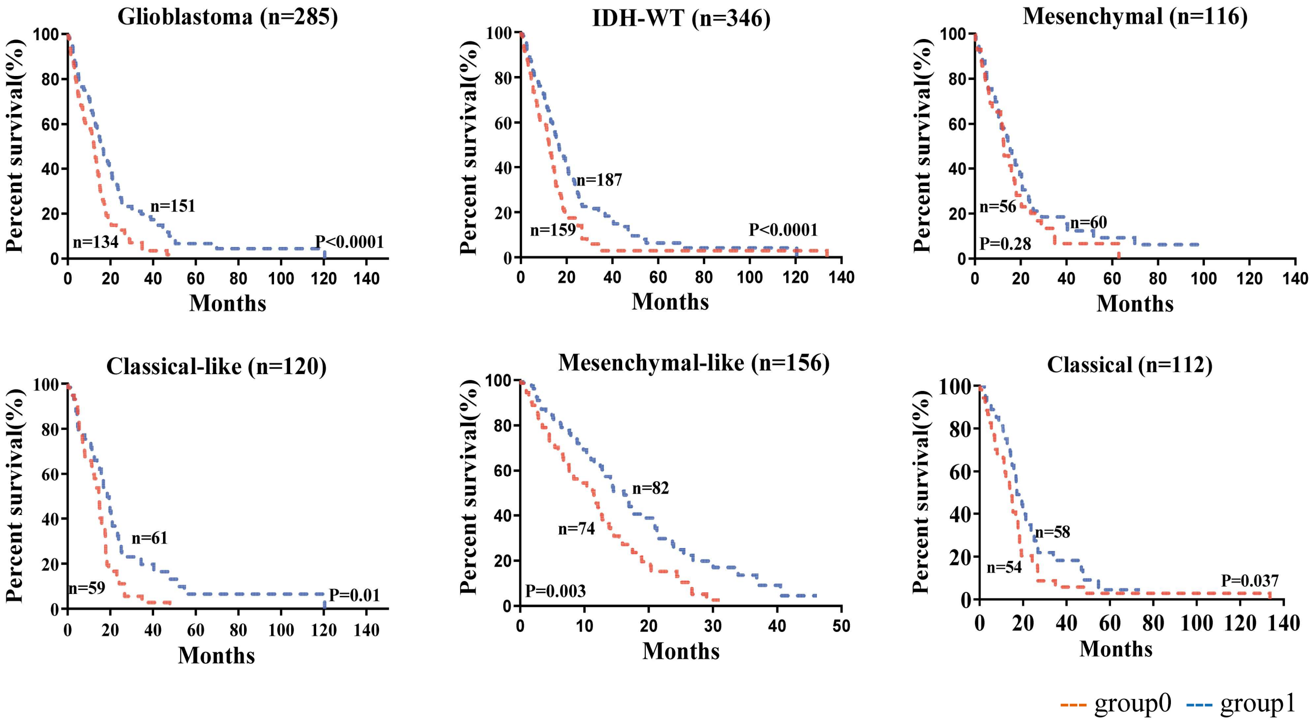

Supplementary Figure S8. Survival of TCGA glioma patients stratified by deep learning model .red line, the deep learning model prediction label is 0; blue line, model prediction label is 1. n, number of patients; p-value was determined using the log-rank test.

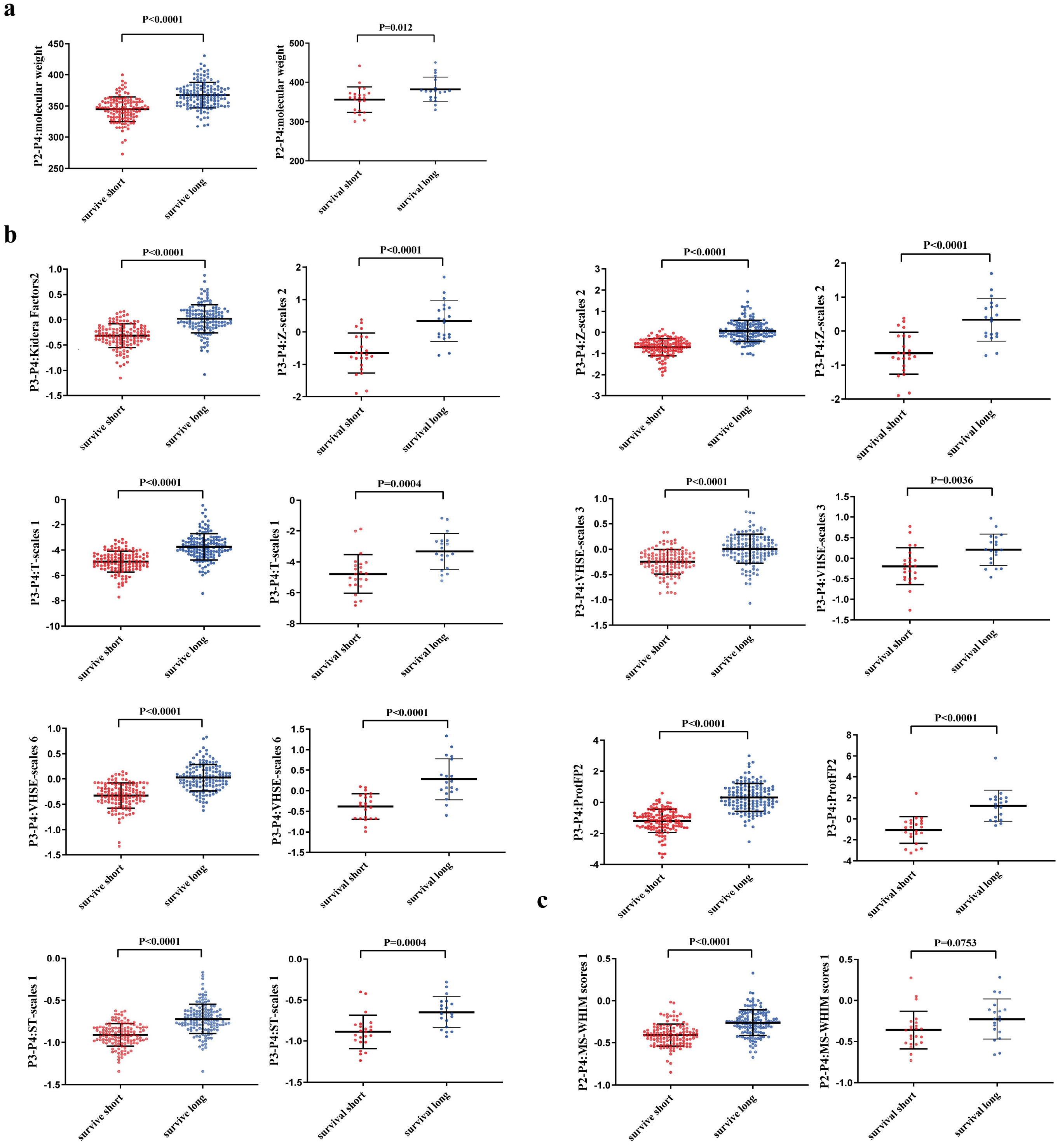

Supplementary Figure S9. Comparison of the similarity of valid feature values between long-term survival and short-term survival groups of IDH wild-type GBM in two cohorts. **a**, molecular weight related features. **b**, molecular size/volume related features. **c**, molecular electrostatic potential/polarity related features. P-value was calculated by unpaired T test.). The left and the right col contain the TCGA cohort and Pri cohort, respectively.

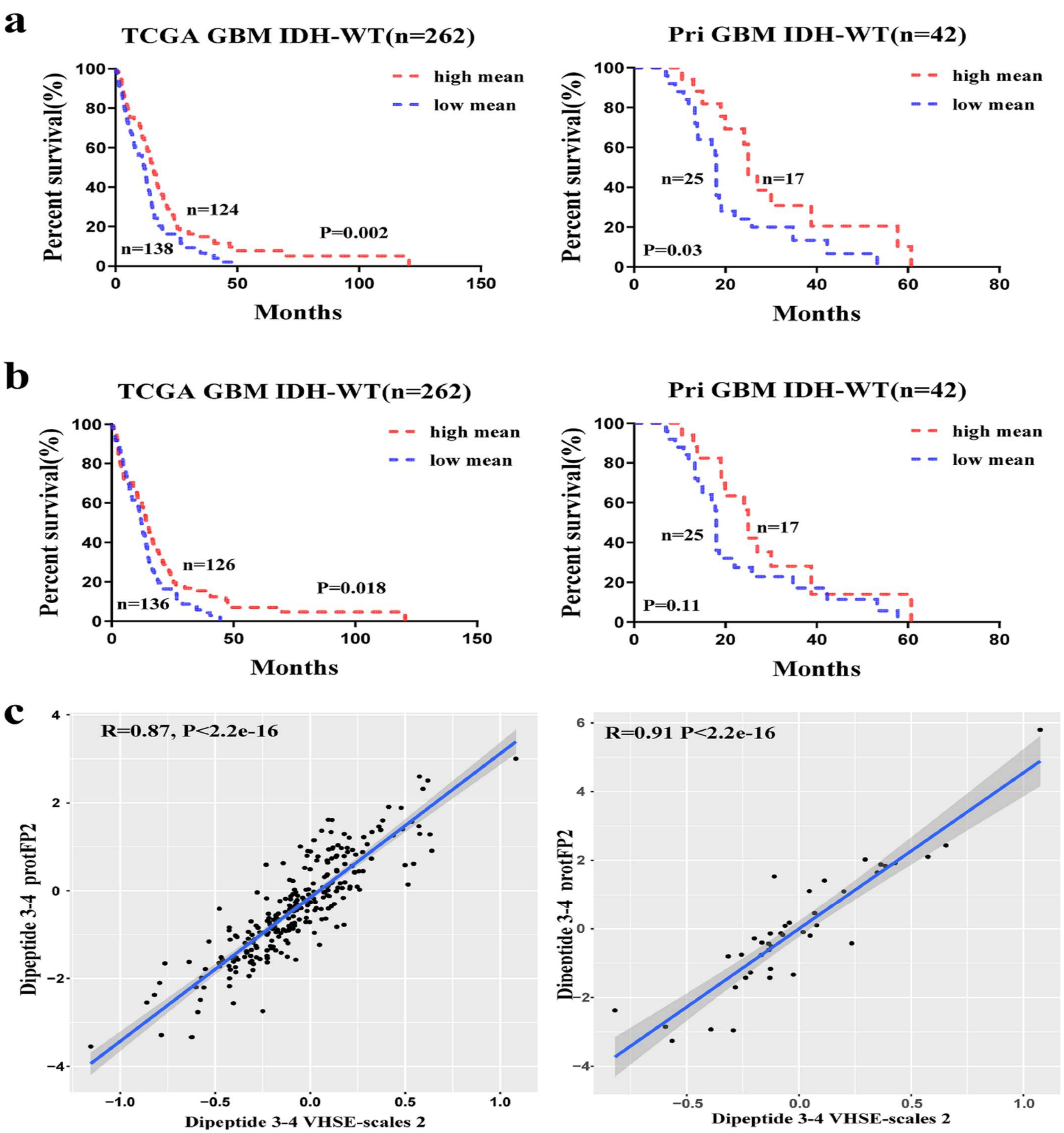

Supplementary Figure S10. Survival of glioma patients stratified according to 2 feature values, and analysis of the correlations between these features in two cohorts. **a**, absolute position 3 and 4 composed-dipeptide protFP2 value ; **b**, absolute position 3 and 4 composed-dipeptide VHSE-scale 2. red line, high mean value, blue , low mean value; n, number of patients; p-value was determined using the log-rank test. **c**, the Pearson correlation coefficient of the two features. The left and the right col contain the TCGA cohort and Pri cohort, respectively.

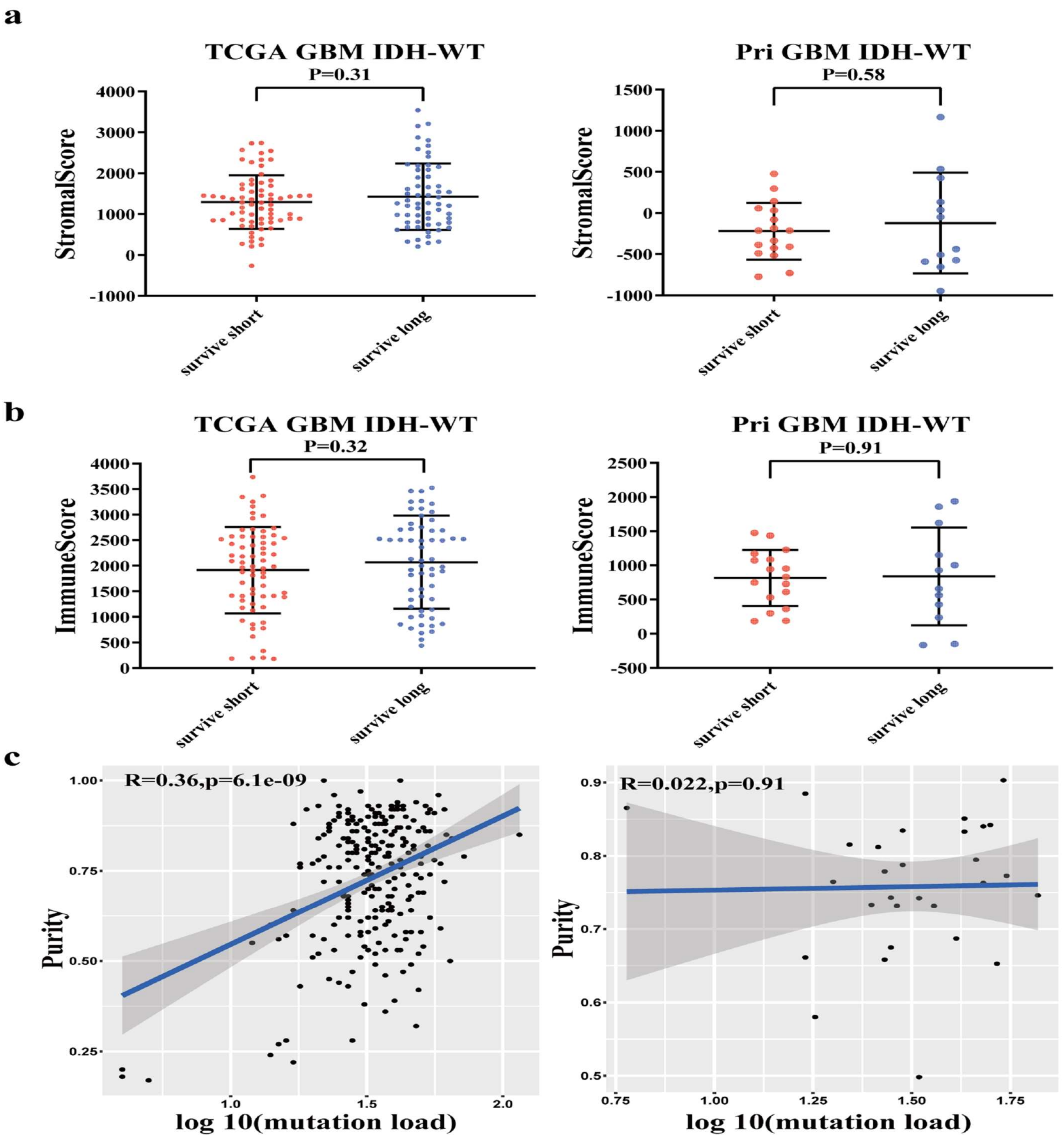

Supplementary Figure S11. Comparison of the similarity of immune score and stromal score between two groups and the correlation analysis between purity and mutation load. **a**, stromal score. **b**, immune score. Immune score and stromal score were derived by ESTIMATE. P value was calculated by unpaired T test. **c**, pearson correlation between purity and log10(mutation load). The left and the right col contain the TCGA cohort and Pri cohort, respectively.

Supplementary Table S1. Multivariate Cox regression analysis including position 3-4 composed-dipeptide VHSE-scale 2 value, mutation load and age for TCGA IDH wild type GBM (n=262).

TCGA cohort (n=262)	HR	95%CI	P
Age	1.0306	[1.0163,1.0452]	2.42e-05
Mutation load	0.9866	[0.9729,1.0005]	0.0595
Dipeptide 3-4 VHSE2	0.5732	[0.3661,0.8972]	0.0149

Supplementary Table S2. Multivariate Cox regression analysis including position 3-4 composed-dipeptide VHSE-scale 2 value, mutation load and age for Pri IDH wild type GBM (n=42).

Pri cohort (n=42)	HR	95%CI	P
Age	1.0230	[0.9914,1.0555]	0.1555
Mutation load	1.0047	[0.9872,1.0226]	0.6011
Dipeptide 3-4 VHSE2	0.3327	[0.1120,0.9885]	0.0476

Supplementary Table S3. Multivariate Cox regression analysis including position 3-4 composed-dipeptide protFP 2 value, mutation load and age for TCGA IDH wild type GBM (n=262).

TCGA cohort (n=262)	HR	95%CI	P
Age	1.0303	[1.0159,1.0449]	3.19e-05
Mutation load	0.9865	[0.9727,1.0005]	0.0579
Dipeptide 3-4 protFP2	0.8818	[0.7804,0.9964]	0.0437

Supplementary Table S4. Multivariate Cox regression analysis including position 3-4 composed-dipeptide protFP 2 value, mutation load and age for Pri IDH wild type GBM (n=42).

Pri cohort (n=42)	HR	95%CI	P
Age	1.024711	[0.9936,1.0568]	0.1208
Mutation load	1.0052	[0.9877,1.0230]	0.5641
Dipeptide 3-4 protFP2	0.7868	[0.6244,0.9915]	0.0421

Supplementary Table S5. Functional annotation for the lists of genes differentially expressed analyzed by GSEA in TCGA cohort.

GENE SET	NAME	SIZE	ES	NES	NOM p-val
NERVOUS SYSTEM DEVELOPMENT	GO_REGULATION_OF_CELL_SIZE	161	-0.3763	-1.5820	0.0200
	GO_CELL_VOLUME_HOMEOSTASIS	26	-0.4998	-1.6130	0.0199
	GO_REGULATION_OF_EXTENT_OF_CELL_GROWTH	98	-0.3821	-1.4872	0.0432
	GO_NEGATIVE_REGULATION_OF_AXONOGENESIS	65	-0.4853	-1.7992	0.0060
	GO_NEGATIVE_REGULATION_OF_CELL_MORPHOGENESIS_INVOLVED_IN_DIFFERENTIATION	114	-0.4008	-1.6149	0.0161
	GO_REGULATION_OF_AXONOGENESIS	163	-0.3987	-1.6032	0.0236
	GO_NEGATIVE_REGULATION_OF_NEURON_DIFFERENTIATION	180	-0.3794	-1.6456	0.0078
	GO_NEGATIVE_REGULATION_OF_NERVOUS_SYSTEM_DEVELOPMENT	247	-0.3620	-1.5871	0.0078
	GO_NEGATIVE_REGULATION_OF_CELL_MORPHOGENESIS_INVOLVED_IN_DIFFERENTIATION	114	-0.4008	-1.6149	0.0161
	GO_NEGATIVE_REGULATION_OF_CELL_DEVELOPMENT	283	-0.3783	-1.7265	0.0000
	GO_REGULATION_OF_GLIAL_CELL_PROLIFERATION	18	-0.5012	-1.5460	0.0427
	GO_REGULATION_OF_NEURON_MIGRATION	27	-0.5358	-1.7249	0.0059
FOREBRAIN DEVELOPMENT	GO_FOREBRAIN_CELL_MIGRATION	58	-0.4223	-1.6291	0.0077
	GO_CEREBRAL_CORTEX_CELL_MIGRATION	39	-0.4377	-1.5670	0.0200
	GO_TELENCEPHALON_DEVELOPMENT	205	-0.3176	-1.3907	0.0454
	GO_TELENCEPHALON_GLIAL_CELL_MIGRATION	17	-0.6088	-1.7113	0.0100
	GO_CEREBRAL_CORTEX_RADIALLY_ORIENTED_CELL_MIGRATION	25	-0.5699	-1.8023	0.0078
GLIAL CELL DEVELOPMENT	GO_GLIAL_CELL_MIGRATION	34	-0.4837	-1.7057	0.0059
	GO_ASTROCYTE_DEVELOPMENT	18	-0.6128	-1.8593	0.0000
	GO_GLIOGENESIS	169	-0.3763	-1.4899	0.0264
	GO_GLIAL_CELL_DEVELOPMENT	73	-0.4118	-1.5100	0.0336
KIDNEY DEVELOPMENT	GO_UROGENITAL_SYSTEM_DEVELOPMENT	287	-0.3096	-1.4206	0.0288
	GO_GLOMERULUS_DEVELOPMENT	46	-0.4705	-1.6512	0.0144
	GO_METANEPHRIC_NEPHRON_MORPHOGENESIS	20	-0.5068	-1.5361	0.0433
	GO_METANEPHRIC_NEPHRON_DEVELOPMENT	31	-0.4970	-1.6176	0.0181
	GO_NEPHRON_DEVELOPMENT	110	-0.3656	-1.5165	0.0335
	GO_RENAL_SYSTEM_VASCULATURE_DEVELOPMENT	18	-0.6440	-1.7302	0.0161
EPIDERMIS DEVELOPMENT	GO_REGULATION_OF_EPITHELIAL_CELL_DIFFERENTIATION	112	-0.3763	-1.5820	0.0200
	GO_NEGATIVE_REGULATION_OF_EPIDERMIS_DEVELOPMENT	15	-0.4998	-1.6130	0.0199
	GO_REGULATION_OF_EPIDERMIS_DEVELOPMENT	59	-0.3821	-1.4872	0.0432
	GO_REGULATION_OF_EPIDERMAL_CELL_DIFFERENTIATION	41	-0.4853	-1.7992	0.0060
	GO_NEGATIVE_REGULATION_OF_EPITHELIAL_CELL_DIFFERENTIATION	34	-0.4008	-1.6149	0.0161
DETECTION OF LIGHT STIMULUS	GO_DETECTION_OF_LIGHT_STIMULUS	52	-0.3987	-1.6032	0.0236
	GO_DETECTION_OF_VISIBLE_LIGHT	37	-0.3794	-1.6456	0.0078
	GO_PHOTOTRANSDUCTION	39	-0.3620	-1.5871	0.0078
	GO_PHOTOTRANSDUCTION_VISIBLE_LIGHT	18	-0.4008	-1.6149	0.0161
	GO_SKELETAL_MUSCLE_CELL_DIFFERENTIATION	49	-0.4770	-1.6848	0.0105
	GO_MUSCLE_TISSUE_DEVELOPMENT	253	-0.3172	-1.4376	0.0370

MUSCLE DEVELOPMENT	GO_CARDIAC_VENTRICLE_MORPHOGENESIS	62	-0.4266	-1.6523	0.0102
	GO_VENTRICULAR_CARDIAC_MUSCLE_TISSUE_DEVELOPMENT	45	-0.4119	-1.4828	0.0408
	GO_CARDIAC_VENTRICLE_DEVELOPMENT	103	-0.3493	-1.4321	0.0459
	GO_CARDIAC_MUSCLE_TISSUE_DEVELOPMENT	130	-0.3393	-1.4517	0.0492
	GO_CARDIAC_CHAMBER_MORPHOGENESIS	103	-0.3637	-1.4518	0.0424
	GO_ATRIOVENTRICULAR_VALVE_DEVELOPMENT	19	-0.5456	-1.5728	0.0269
	GO_ATRIOVENTRICULAR_VALVE_MORPHOGENESIS	16	-0.5544	-1.5179	0.0430
	GO_OUTFLOW_TRACT_MORPHOGENESIS	56	-0.4390	-1.6306	0.0187
NEGATIVE REGULATION OF ANGIOGENESIS	GO_NEGATIVE_REGULATION_OF_BLOOD_VESSEL_ENDOTHELIAL_CELL_MIGRATION	24	-0.3791	-1.6270	0.0103
	GO_NEGATIVE_REGULATION_OF_EPITHELIAL_CELL_MIGRATION	53	-0.5702	-1.5534	0.0478
	GO_NEGATIVE_REGULATION_OF_ENDOTHELIAL_CELL_MIGRATION	39	-0.3559	-1.4293	0.0400
	GO_NEGATIVE_REGULATION_OF_VASCULATURE_DEVELOPMENT	79	-0.4178	-1.5265	0.0346
ENDOTHELIUM DEVELOPMENT	GO_MORPHOGENESIS_OF_AN_ENDOTHELIUM	16	-0.5385	-1.8107	0.0021
	GO_ENDOTHELIUM_DEVELOPMENT	84	-0.5350	-1.6806	0.0250
	GO_ENDOTHELIAL_CELL_DIFFERENTIATION	67	-0.4050	-1.5413	0.0268
	GO_ENDOTHELIAL_CELL_DEVELOPMENT	43	-0.4584	-1.6089	0.0335
LIPID KINASE ACTIVITY	GO_REGULATION_OF_PHOSPHOLIPID_METABOLIC_PROCESS	57	-0.3955	-1.5765	0.0247
	GO_REGULATION_OF_LIPID_KINASE_ACTIVITY	45	-0.6210	-1.7043	0.0185
	GO_REGULATION_OF_PHOSPHATIDYLINOSITOL_3_KINASE_ACTIVITY	37	-0.4070	-1.5577	0.0312
	GO_POSITIVE_REGULATION_OF_LIPID_KINASE_ACTIVITY	30	-0.4084	-1.5323	0.0271
NEGATIVE REGULATION OF ENDOCYTOSIS	GO_REGULATION_OF_RECEPTOR_INTERNALIZATION	37	-0.4643	-1.5897	0.0374
	GO_REGULATION_OF_RECEPTOR_MEDIATED_ENDOCYTOSIS	76	-0.4205	-1.5133	0.0452
	GO_NEGATIVE_REGULATION_OF_RECEPTOR_MEDIATED_ENDOCYTOSIS	17	-0.4623	-1.6021	0.0245
	GO_NEGATIVE_REGULATION_OF_ENDOCYTOSIS	38	-0.5463	-1.8176	0.0082
CELL CYCLE	GO_REGULATION_OF_MEIOTIC_CELL_CYCLE	37	-0.5633	-1.8370	0.0041
	GO_REGULATION_OF_REPRODUCTIVE_PROCESS	119	-0.4719	-1.6415	0.0144
	GO_POSITIVE_REGULATION_OF_REPRODUCTIVE_PROCESS	51	-0.4182	-1.6087	0.0167
	GO_POSITIVE_REGULATION_OF_MULTI_ORGANISM_PROCESS	148	-0.6680	-2.0132	0.0022
	GO_REGULATION_OF_MEIOTIC_NUCLEAR_DIVISION	26	-0.4526	-1.5995	0.0276
	GO_POSITIVE_REGULATION_OF_CELL_CYCLE	305	-0.4041	-1.5413	0.0303
	GO_NEGATIVE_REGULATION_OF_CELL_CYCLE_ARREST	17	-0.5100	-1.8460	0.0039
	GO_POSITIVE_REGULATION_OF_CELL_DIVISION	119	-0.4054	-1.4644	0.0321
	GO_POSITIVE_REGULATION_OF_NUCLEAR_DIVISION	59	-0.6066	-1.8365	0.0060
ACTIN AND CELL BEHAVIOR	GO_ACTOMYOSIN	53	-0.4592	-1.5851	0.0148
	GO_POSITIVE_REGULATION_OF_LAMELLIPODIUM_ORGANIZATION	21	-0.3330	-1.4821	0.0149
	GO_ACTIN_FILAMENT_BUNDLE	48	-0.4802	-1.7601	0.0083
	GO_ACTIN_CYTOSKELETON	400	-0.3408	-1.5084	0.0203
	GO_MYOFILAMENT	22	-0.5122	-1.6343	0.0191
	GO_ACTIN_BINDING	362	-0.3071	-1.3971	0.0472
	GO_FILAMENTOUS_ACTIN	16	-0.5672	-1.6450	0.0273
	GO_REGULATION_OF_ACTIN_FILAMENT_DEPOLYMERIZATION	46	-0.3676	-1.6096	0.0061
	GO_POSITIVE_REGULATION_OF_PROTEIN_DEPOLYMERIZATION	18	-0.4140	-1.6294	0.0121
	GO_POSITIVE_REGULATION_OF_PROTEIN_COMPLEX_DISASSEMBLY	24	-0.4813	-1.6042	0.0459

Supplementary Table S6. Functional annotation for the lists of genes differentially expressed analyzed by GSEA in Pri cohort.

GENE SET	NAME	SIZE	ES	NES	NOM p-val
DEVELOPMENT	GO_NEPHRON_EPITHELIUM_DEVELOPMENT	93	-0.4751	-1.6299	0.0106
	GO_NEPHRON_DEVELOPMENT	115	-0.4694	-1.5868	0.0298
	GO_BRANCHING_INVOLVED_IN_URETERIC_BUD_MORPHOGENESIS	44	-0.5077	-1.6568	0.0108
	GO_REGULATION_OF_CELL_PROLIFERATION_INVOLVED_IN_HEART_MORPHOGENESIS	15	-0.6520	-1.6017	0.0308
	GO_MESONEPHRIC_TUBULE_MORPHOGENESIS	53	-0.4865	-1.6281	0.0168
	GO_METANEPHRIC_NEPHRON_DEVELOPMENT	32	-0.5624	-1.6702	0.0261
	GO_MESENCHYMAL_TO_EPITHELIAL_TRANSITION	15	-0.5699	-1.5512	0.0306
	GO_REGULATION_OF_MESONEPHROS_DEVELOPMENT	26	-0.5620	-1.6528	0.0063
	GO_METANEPHROS_MORPHOGENESIS	28	-0.4907	-1.4784	0.0273
	GO_REGULATION_OF_MORPHOGENESIS_OF_A_BRANCHING_STRUCTURE	53	-0.5160	-1.7473	0.0021
	GO_KIDNEY_MORPHOGENESIS	82	-0.4616	-1.5773	0.0068
	GO_METANEPHROS_DEVELOPMENT	81	-0.4828	-1.6600	0.0022
	GO_POSITIVE_REGULATION_OF_KIDNEY_DEVELOPMENT	41	-0.4881	-1.5694	0.0107
	GO_POSITIVE_REGULATION_OF_MESONEPHROS_DEVELOPMENT	22	-0.5434	-1.5730	0.0145
	GO_RENAL_TUBULE_DEVELOPMENT	78	-0.4704	-1.5806	0.0218
	GO_DEVELOPMENTAL_INDUCTION	27	-0.5744	-1.7504	0.0022
	GO_MESONEPHROS_DEVELOPMENT	90	-0.4135	-1.4647	0.0371
	GO_KIDNEY_EPITHELIUM_DEVELOPMENT	125	-0.4409	-1.5676	0.0149
	GO_KIDNEY_MESENCHYME_DEVELOPMENT	17	-0.6525	-1.7126	0.0168
	GO_REGULATION_OF_ORGAN_FORMATION	32	-0.5590	-1.8287	0.0000
	GO_REGULATION_OF_HEART_MORPHOGENESIS	29	-0.5182	-1.5953	0.0165
	GO_ORGAN_INDUCTION	16	-0.6573	-1.8843	0.0000
	GO_SMOOTH_MUSCLE_CELL_DIFFERENTIATION	30	-0.6063	-1.6321	0.0193
	GO_MUSCLE_CELL_DIFFERENTIATION	232	-0.3789	-1.4548	0.0445
	GO_POSITIVE_REGULATION_OF_CARDIAC_MUSCLE_CELL_PROLIFERATION	19	-0.5747	-1.6615	0.0143
	GO_MUSCLE_STRUCTURE_DEVELOPMENT	422	-0.3797	-1.4761	0.0297
	GO_MYOFILAMENT	24	-0.4826	-1.4807	0.0494
	GO_MUSCLE_ORGAN_DEVELOPMENT	268	-0.3722	-1.4283	0.0398
	GO_REGULATION_OF_CARDIAC_MUSCLE_CELL_PROLIFERATION	29	-0.4985	-1.5391	0.0186
	GO_REGULATION_OF_HEART_GROWTH	42	-0.4168	-1.4310	0.0319
	GO_SKELETAL_MUSCLE_ORGAN_DEVELOPMENT	131	-0.3886	-1.4271	0.0394
	GO_NEGATIVE_REGULATION_OF_EPITHELIAL_CELL_DIFFERENTIATION	37	-0.5377	-1.7288	0.0000
	GO_NEGATIVE_REGULATION_OF_EPIDERMIS_DEVELOPMENT	16	-0.5956	-1.6537	0.0063
	GO_REGULATION_OF_EPIDERMIS_DEVELOPMENT	63	-0.4213	-1.4555	0.0227
	GO_REGULATION_OF_EPIDERMAL_CELL_DIFFERENTIATION	45	-0.5144	-1.6747	0.0082
	GO_EMBRYONIC_SKELETAL_SYSTEM_MORPHOGENESIS	93	-0.5174	-1.6215	0.0201
	GO_EMBRYONIC_SKELETAL_SYSTEM_DEVELOPMENT	121	-0.4927	-1.5897	0.0308
	GO_EMBRYONIC_FORELIMB_MORPHOGENESIS	32	-0.6312	-1.8401	0.0022
	GO_FORELIMB_MORPHOGENESIS	40	-0.6591	-2.0074	0.0021
	GO_HINDLIMB_MORPHOGENESIS	37	-0.5516	-1.6244	0.0258

CELL FATE	GO_MUSCLE_CELL_FATE_COMMITMENT	15	-0.6617	-1.7801	0.0103
	GO_ECTODERMAL_PLACODE_DEVELOPMENT	15	-0.5624	-1.4811	0.0406
	GO_NEURON_FATE_COMMITMENT	67	-0.4510	-1.4405	0.0488
	GO_CELL_FATE_COMMITMENT_INVOLVED_IN_FORMATION_OF_PRIMARY_GERM_LAYER	28	-0.4790	-1.4892	0.0471
	GO_CELL_FATE_COMMITMENT	226	-0.3922	-1.4485	0.0489
DNA TRANSCRIPTION	GO_NUCLEOSOMAL_DNA_BINDING	30	-0.5613	-1.5492	0.0398
	GO_ENHANCER_BINDING	92	-0.5125	-1.7069	0.0000
	GO_RNA_POLYMERASE_II_DISTAL_ENHANCER_SEQUENCE_SPECIFIC_DNA_BINDING	64	-0.5469	-1.7511	0.0020
	GO_TRANSCRIPTION_FACTOR_ACTIVITY_RNA_POLYMERASE_II_DISTAL_ENHANCER_SEQUENCE_SPECIFIC_BINDING	89	-0.4780	-1.5828	0.0295
	GO_TRANSCRIPTIONAL_ACTIVATOR_ACTIVITY_RNA_POLYMERASE_II_CORE_PROMOTER_PROXIMAL_REGION_SEQUENCE_SPECIFIC_BINDING	226	-0.4546	-1.6407	0.0088
	GO_TRANSCRIPTIONAL_ACTIVATOR_ACTIVITY_RNA_POLYMERASE_II_TRANSCRIPTION_REGULATORY_REGION_SEQUENCE_SPECIFIC_BINDING	314	-0.4375	-1.6303	0.0065
	GO_TRANSCRIPTION_FACTOR_ACTIVITY_RNA_POLYMERASE_II_CORE_PROMOTER_PROXIMAL_REGION_SEQUENCE_SPECIFIC_BINDING	326	-0.4101	-1.5091	0.0441
	GO_CORE_PROMOTER_PROXIMAL_REGION_DNA_BINDING	363	-0.4048	-1.5120	0.0338
	GO_HMG_BOX_DOMAIN_BINDING	18	-0.5867	-1.5328	0.0419
CELL DIFFERENCIATION AND PATTERN FORMATION	GO_PROXIMAL_DISTAL_PATTERN_FORMATION	32	-0.5980	-1.8268	0.0022
	GO_ANTERIOR_POSTERIOR_PATTERN_SPECIFICATION	194	-0.4535	-1.5828	0.0317
	GO_ANTERIOR_POSTERIOR_AXIS_SPECIFICATION	48	-0.4958	-1.5677	0.0229
	GO_EMBRYONIC_PATTERN_SPECIFICATION	58	-0.4441	-1.5373	0.0185
	GO_AXIS_SPECIFICATION	90	-0.4567	-1.5399	0.0235
AA BIOSYNTHETIC AND METABOLIC	GO_METHIONINE_METABOLIC_PROCESS	18	-0.6747	-1.7151	0.0040
	GO_SULFUR_AMINO_ACID_BIOSYNTHETIC_PROCESS	19	-0.5899	-1.5468	0.0413
	GO_ASPARTATE_FAMILY_AMINO_ACID_BIOSYNTHETIC_PROCESS	23	-0.5939	-1.5312	0.0276
	GO_SULFUR_AMINO_ACID_METABOLIC_PROCESS	40	-0.5547	-1.6136	0.0079
PHOSPHATIDYLCHOLINE METABOLIC	GO_LYSOPHOSPHOLIPASE_ACTIVITY	20	-0.5709	-1.6967	0.0042
	GO_PHOSPHOLIPASE_A2_ACTIVITY	31	-0.5016	-1.6308	0.0121
	GO_PHOSPHATIDYLETHANOLAMINE_ACYL_CHAIN_REMODELING	23	-0.5646	-1.6702	0.0041
	GO_PHOSPHATIDYLSERINE_METABOLIC_PROCESS	28	-0.4944	-1.5093	0.0265
	GO_PHOSPHATIDYLCHOLINE_ACYL_CHAIN_REMODELING	26	-0.4962	-1.5195	0.0244
	GO_PHOSPHATIDYLCHOLINE_ACYL_CHAIN_REMODELING	26	-0.4962	-1.5195	0.0244
DNA CATABOLIC	GO_APOPTOTIC_DNA_FRAGMENTATION	15	-0.6423	-1.6850	0.0084
	GO_DNA_CATABOLIC_PROCESS_ENDONUCLEOLYTIC	19	-0.6155	-1.6531	0.0167
	GO_DNA_CATABOLIC_PROCESS	27	-0.5160	-1.4684	0.0354
	GO_NUCLEASE_ACTIVITY	196	-0.4428	-1.5150	0.0438
	GO_ENDODEOXYRIBONUCLEASE_ACTIVITY	49	-0.5508	-1.7097	0.0083
	GO_DEOXYRIBONUCLEASE_ACTIVITY	65	-0.5413	-1.6832	0.0042
	GO_EXONUCLEASE_ACTIVITY	76	-0.5044	-1.5316	0.0493
	GO_EXODEOXYRIBONUCLEASE_ACTIVITY	15	-0.6494	-1.5721	0.0285
	GO_ENDONUCLEASE_ACTIVITY_ACTIVE_WITH_EITHER_RIBO_OR_DEOXYRIBONUCLEIC_ACIDS_AND_PRODUCING_3_PHOSPHOMONOESTERS	19	-0.5400	-1.4735	0.0432

Supplementary Table S7. Compare the effects with others methods.

Model	AUC(TCGA)	P-value(KM)
**neoDL**	0.988	1.65E-07
**Random Forest**	0.963	1.08E-07
**SVM**	0.952	7.4E-06

Supplementary Table S8. total features.

Features	Calculated site		
**Physical - chemical properties**			
**hydrophobicity index; hydrophobic moment; theoretical net charge ; molecular weight ; Boman (Potential Protein Interaction) index; aliphatic index**	the mutant peptide;Mutation position<br>the absolute site 1/2/3/4/5/6/7/8/9 of the mutant peptide<br>dipeptide composed of the mutation position and the previous one of the mutant peptide<br>tripeptide consisting of the mutation position and the first two of the mutation peptide<br>tripeptide consisting of the mutated position and the last two of the mutated peptide;<br>dipeptide composed of the mutation position and the latter of the mutant peptide;<br>tripeptide consisting of one bit before to one bit after the mutation of the mutant peptide<br>changes at mutant positions during the mutation	site 1and 2 compose-dipeptide of the mutant peptide<br>site 2 and 3 compose-dipeptide of the mutant peptide<br>site 3 and 4 compose-dipeptide of the mutant peptide<br>site 4 and 5 compose-dipeptide of the mutant peptide<br>site 5 and 6 compose-dipeptide of the mutant peptide<br>site 6 and 7 compose-dipeptide of the mutant peptide<br>site 7 and 8 compose-dipeptide of the mutant peptide<br>site 8 and 9 compose-dipeptide of the mutant peptide	site 1 to 3 compose-tripeptide of the mutant peptide<br>site 2 to 4 compose-tripeptide of the mutant peptide<br>site 3 to 5 compose-tripeptide of the mutant peptide<br>site 4 to 6 compose-tripeptide of the mutant peptide<br>site 5 to 7 compose-tripeptide of the mutant peptide<br>site 6 to 8 compose-tripeptide of the mutant peptide<br>site 7 to 9 compose-tripeptide of the mutant peptide<br>changes during mutation
auto-correlation index ; auto-covariance index ; cross-covariance index	the mutant peptide;<br>changes during mutation		
instability index	the mutant peptide;<br>changes during mutation<br>tripeptide consisting of the mutated position and the last two of the mutated peptide<br>tripeptide consisting of the mutation position and the first two of the mutation peptide	site 1 to 3 compose-tripeptide of the mutant peptide<br>site 5 to 7 compose-tripeptide of the mutant peptide<br>site 6 to 8 compose-tripeptide of the mutant peptide<br>site 7 to 9 compose-tripeptide of the mutant peptide	site 2 to 4 compose-tripeptide of the mutant peptide<br>site 3 to 5 compose-tripeptide of the mutant peptide<br>site 4 to 6 compose-tripeptide of the mutant peptide
aaComp			
aaComp :Tiny ;Small ;Aliphatic ;Aromatic;Nonpolar ;Polar ;Charged ;Basic ;Acidic	the mutant peptide;<br>dipeptide composed of the mutation position and the latter of the mutant peptide<br>tripeptide consisting of the mutated position and the last two of the mutated peptide<br>tripeptide consisting of the mutation position and the first two of the mutation peptide<br>changes during mutation<br>Mutation position<br>changes at mutant positions during the mutation<br>dipeptide composed of the mutation position and the previous one of the mutant peptide<br>tripeptide consisting of one bit before to one bit after the mutation of the mutant peptide	site 1and 2 compose-dipeptide of the mutant peptide<br>site 2 and 3 compose-dipeptide of the mutant peptide<br>site 3 and 4 compose-dipeptide of the mutant peptide<br>site 4 and 5 compose-dipeptide of the mutant peptide<br>site 5 and 6 compose-dipeptide of the mutant peptide<br>site 6 and 7 compose-dipeptide of the mutant peptide<br>site 7 and 8 compose-dipeptide of the mutant peptide<br>site 8 and 9 compose-dipeptide of the mutant peptide	site 1 to 3 compose-tripeptide of the mutant peptide<br>site 2 to 4 compose-tripeptide of the mutant peptide<br>site 3 to 5 compose-tripeptide of the mutant peptide<br>site 4 to 6 compose-tripeptide of the mutant peptide<br>site 5 to 7 compose-tripeptide of the mutant peptide<br>site 6 to 8 compose-tripeptide of the mutant peptide<br>site 7 to 9 compose-tripeptide of the mutant peptide<br>the absolute site 1/2/3/4/5/6/7/8/9 of the mutant peptide
aaComp: change	Tiny->small at mutant position during mutation	small->Tiny at mutant position during mutation	Aliphatic->Aromatic at mutant position during mutation
	Polar ->Nonpolar at mutant position during mutation	Basic -> Acidic at mutant position during mutation	Acidic ->Basic at mutant position during mutation
	Nonpolar -> Polar at mutant position during mutation	Aromatic->Aliphatic at mutant position during mutation	
66 descriptors for amino acid			
Cruciani properties;<br>Kidera factors;<br>Z-scales;<br>FASGAI vectors ;<br>T-scales'<br>VHSE-scales ;<br>protFP descriptors;<br>ST-scales;<br>BLOSUM62 derived indices ;<br>MS-WHIM scores	the mutant peptide;<br>Mutation position<br>dipeptide composed of the mutation position and the previous one of the mutant peptide<br>dipeptide composed of the mutation position and the latter of the mutant peptide<br>tripeptide consisting of the mutation position and the first two of the mutation peptide<br>tripeptide consisting of the mutated position and the last two of the mutated peptide<br>the absolute site 1/2/3/4/5/6/7/8/9 of the mutant peptide<br>tripeptide consisting of one bit before to one bit after the mutation of the mutant peptide<br>changes during mutation	site 1and 2 compose-dipeptide of the mutant peptide<br>site 2 and 3 compose-dipeptide of the mutant peptide<br>site 3 and 4 compose-dipeptide of the mutant peptide<br>site 4 and 5 compose-dipeptide of the mutant peptide<br>site 5 and 6 compose-dipeptide of the mutant peptide<br>site 6 and 7 compose-dipeptide of the mutant peptide<br>site 7 and 8 compose-dipeptide of the mutant peptide<br>site 8 and 9 compose-dipeptide of the mutant peptide<br>changes at mutant positions during the mutation	site 1 to 3 compose-tripeptide of the mutant peptide<br>site 2 to 4 compose-tripeptide of the mutant peptide<br>site 3 to 5 compose-tripeptide of the mutant peptide<br>site 4 to 6 compose-tripeptide of the mutant peptide<br>site 5 to 7 compose-tripeptide of the mutant peptide<br>site 6 to 8 compose-tripeptide of the mutant peptide<br>site 7 to 9 compose-tripeptide of the mutant peptide

Features	Calculated site			
Amino acid composition				
Amino Acid type: 20 amino acid type	Mutation position	The first two positions of the mutant peptide	The middle three positions of the mutant peptide	the first position of the mutant peptide
	The first three positions of the mutant peptide	The last two positions of the mutant peptide	The last three positions of the mutant peptide	the last position of the mutant peptide
Entropy	the mutant peptide;			
	dipeptide composed of the mutation position and the previous one of the mutant peptide	tripeptide consisting of the mutated position and the last two of the mutated peptide	dipeptide composed of the mutation position and the latter of the mutant peptide	
	tripeptide consisting of one bit before to one bit after the mutation of the mutant peptide	tripeptide consisting of the mutation position and the first two of the mutation peptide	changes during mutant process	
entropyseq	Amino Acid Type(20） of wild type peptide	changes of tripeptide consisting of the mutated position and the last two of the mutated peptide during mutation	site 1and 2 compose-dipeptide of the mutant peptide	site 4 and 5 compose-dipeptide of the mutant peptide
	Amino Acid Type(20） of mutant type peptide	changes of tripeptide consisting of the mutation position and the first two of the mutation peptide during mutation	site 2 and 3 compose-dipeptide of the mutant peptide	site 5 and 6 compose-dipeptide of the mutant peptide
	changes of dipeptide composed of the mutation position and the previous one of the mutant peptide during mutation	changes of dipeptide composed of the mutation position and the latter of the mutant peptide during mutation	site 3 and 4 compose-dipeptide of the mutant peptide	site 6 and 7 compose-dipeptide of the mutant peptide
			site 7 and 8 compose-dipeptide of the mutant peptide	site 8 and 9 compose-dipeptide of the mutant peptide
Mutation position in the top three				
Mutation position in the middle three positions				
Mutation position in the last three positions				

Supplementary Table S9. 189 valid features.

Feature annotation
auto-correlation index of mutant peptide
auto-covariance index of mutant peptide
molecular weight of the dipeptide composed of the mutation position and the previous one of the mutant peptide
instability index of tripeptide consisting of the mutation position and the first two digits of the mutant peptide
hydrophobicity index at the absolute site 4 of the mutant peptide
Hydrophobicity index of site 3 and 4 compose-dipeptide of the mutant peptide
molecular weight of site 3 and 4 compose-dipeptide of the mutant peptide
molecular weight of site 2 to 4 compose-tripeptide of the mutant peptide
Boman (Potential Protein Interaction) index at the absolute site 4 of the mutant peptide
Boman (Potential Protein Interaction) index of site 3 and 4 compose-dipeptide of the mutant peptide
aliphatic index at the absolute site 4 of the mutant peptide
aliphatic index of site 3 and 4 compose-dipeptide of the mutant peptide
aliphatic index of site 6 and 7 compose-dipeptide of the mutant peptide
aliphatic index of site 5 to 7 compose-tripeptide of the mutant peptide
aliphatic index of site 6 to 8 compose-tripeptide of the mutant peptide
The tripeptide composed of site 2 to 4 of the mutant peptide contains Aliphatic amino acids number
The tripeptide composed of site 2 to 4 of the mutant peptide contains Nonpolar amino acids number
The tripeptide composed of site 2 to 4 of the mutant peptide contains Polar amino acids number
The tripeptide composed of site 3 to 5 of the mutant peptide contains Nonpolar amino acids number
The tripeptide composed of site 3 to 5 of the mutant peptide contains Polar amino acids number
The tripeptide composed of site 5 to 7 of the mutant peptide contains Aromatic amino acids number
The tripeptide composed of site 5 to 7 of the mutant peptide contains Basic amino acids number
FASGAI vectors F3 value at the mutant position of mutant peptide
T-scales T1 value at the mutant position of mutant peptide
VHSE-scales VHSE2 value at the mutant position of mutant peptide
protFP descriptors protFP5 at the mutant position of mutant peptide
ST-scales ST1 value at the mutant position of mutant peptide
BLOSUM62 derived indices BLOSUM4 at the mutant position of mutant peptide
Cruciani properties PP2 value of the dipeptide composed of the mutation position and the previous one of the mutant peptide
Kidera factors KF2 value of the dipeptide composed of the mutation position and the previous one of the mutant peptide
FASGAI vectors F3 value value of the dipeptide composed of the mutation position and the previous one of the mutant peptide
T-scales T1 value of the dipeptide composed of the mutation position and the previous one of the mutant peptide
VHSE-scales VHSE2 value of the dipeptide composed of the mutation position and the previous one of the mutant peptide
VHSE-scales VHSE3 value of the dipeptide composed of the mutation position and the previous one of the mutant peptide
ST-scales ST1 value of the dipeptide composed of the mutation position and the previous one of the mutant peptide
BLOSUM62 derived indices BLOSUM4 value of the dipeptide composed of the mutation position and the previous one of the mutant peptide
VHSE-scales VHSE2 value of the dipeptide composed of the mutation position and the latter of the mutant peptide
ST-scales ST4 value of the dipeptide composed of the mutation position and the latter of the mutant peptide
BLOSUM62 derived indices BLOSUM4 value of the dipeptide composed of the mutation position and the latter of the mutant peptide
BLOSUM62 derived indices BLOSUM7 value of the dipeptide composed of the mutation position and the latter of the mutant peptide
VHSE-scales VHSE2 value of the tripeptide consisting of the mutation position and the first two of the mutation peptide
BLOSUM62 derived indices BLOSUM4 value of the tripeptide consisting of the mutation position and the first two of the mutation peptide
BLOSUM62 derived indices BLOSUM9 value of the tripeptide consisting of the mutation position and the first two of the mutation peptide
ST-scales ST8 value for tripeptide consisting of the mutated position and the last two of the mutated peptide
BLOSUM62 derived indices BLOSUM7 value or tripeptide consisting of the mutated position and the last two of the mutated peptide
changes in the number of Aromatic amino acids during the mutation process
changes in the number of Aromatic amino acids at the mutation site during mutation process

The dipeptide composed of site 3 and 4 of the mutant peptide contains Alipahtic amino acids number
The dipeptide composed of site 3 and 4 of the mutant peptide contains Nonpolar amino acids number
The dipeptide composed of site 3 and 4 of the mutant peptide contains Polar amino acids number
The dipeptide composed of site 4 and 5 of the mutant peptide contains Alipahtic amino acids number
The dipeptide composed of site 4 and 5 of the mutant peptide contains Nonpolar amino acids number
The dipeptide composed of site 4 and 5 of the mutant peptide contains Polar amino acids number
The dipeptide composed of site 6 and 7 of the mutant peptide contains Alipahtic amino acids number
The dipeptide composed of site 7 and 8 of the mutant peptide contains Aromatic amino acids number
The dipeptide composed of site 7 and 8 of the mutant peptide contains Basic amino acids number
Mutant peptide with Alipahtic amino acid at position 4
Mutant peptide with Nonpolar amino acid at position 4
Mutant peptide with Polar amino acid at position 4
Mutant peptide with Charge amino acid at position 4
Mutant peptide with Small amino acid at position 5
Mutant peptide with Aromatic amino acid at position 5
Mutant peptide with Basic amino acid at position 5
Mutant peptide with Basic amino acid at position 7
The mutation position of the mutant peptide is W amino acid
The first three positions of the mutant peptide are present F amino acids
The middle three positions of the mutant peptide are present A amino acids
The first two presence of mutant peptides are present F amino acids
The first position of the mutant peptide is the F amino acid
Changes of Kidera factors KF4 value during mutation
Changes of Z-scales Z1 value during mutation
Changes of FASGAI vectors F3 value during mutation
Changes of VHSE-scales VHSE1 value during mutation
Changes of BLOSUM62 derived indices BLOSUM1 value during mutation
Changes in Kidera factor KF4 values at mutant positions during the mutation
Changes of Z-scale Z1 value at mutant positions during the mutation
changes of FASGAI vectors F3 value at mutant positions during the mutation
Change of VHSE-scales VHSE1 value at mutant positions during the mutation
Changes of BLOSUM62 derived indices BLOSUM1 value at mutant positions during the mutation
BLOSUM62 derived indices BLOSUM4 value of the tripeptide consisting of one bit before to one bit after the mutation of the mutant peptide
BLOSUM62 derived indices BLOSUM7 value of the tripeptide consisting of one bit before to one bit after the mutation of the mutant peptide
Entropy of a residue type of amino acid N in wildtype peptide
Entropy of a residue type of amino acid W in wildtype peptide
protFP descriptors protFP8 value for tripeptide consisting of the mutated position and the last two of the mutated peptide
BLOSUM62 derived indices BLOSUM4 value at the absolute site 2 of the mutant peptide
Cruciani properties PP3 value at the absolute site 3 of the mutant peptide
Z-scales Z2 value at the absolute site 3 of the mutant peptide
VHSE-scales VHSE1 value at the absolute site 3 of the mutant peptide
ST-scales ST4 value at the absolute site 3 of the mutant peptide
BLOSUM62 derived indices BLOSUM7 value at the absolute site 3 of the mutant peptide
Cruciani properties PP1 value at the absolute site 4 of the mutant peptide
Kidera factors KF4 value at the absolute site 4 of the mutant peptide
Z-scales Z4 value at the absolute site 4 of the mutant peptide
Z-scales Z2 value at the absolute site 4 of the mutant peptide
FASGAI vector F4 value at the absolute site 4 of the mutant peptide
T-scales T4 value at the absolute site 4 of the mutant peptide

VHSE-scales VHSE1 value at the absolute site 4 of the mutant peptide
VHSE-scales VHSE2 value at the absolute site 4 of the mutant peptide
VHSE-scales VHSE6 value at the absolute site 4 of the mutant peptide
protFP descriptors protFP2 value at the absolute site 4 of the mutant peptide
protFP descriptors protFP8 value at the absolute site 4 of the mutant peptide
ST-scales ST4 value at the absolute site 4 of the mutant peptide
ST-scales ST5 value at the absolute site 4 of the mutant peptide
BLOSUM62 derived indices BLOSUM7 value at the absolute site 4 of the mutant peptide
Z-scales Z4 value at the absolute site 5 of the mutant peptide
FASGAI vector F6 value at the absolute site 5 of the mutant peptide
VHSE-scales VHSE5 value at the absolute site 5 of the mutant peptide
ST-scales ST3 value at the absolute site 5 of the mutant peptide
BLOSUM62 derived indices BLOSUM9 value at the absolute site 5 of the mutant peptide
VHSE-scales VHSE5 value at the absolute site 6 of the mutant peptide
Cruciani properties PP3 value at the absolute site 7 of the mutant peptide
Kidera factors KF10 value at the absolute site 7 of the mutant peptide
T-scales T5 value at the absolute site 7 of the mutant peptide
VHSE-scales VHSE6 value at the absolute site 7 of the mutant peptide
BLOSUM62 derived indices BLOSUM5 value at the absolute site 7 of the mutant peptide
Kidera factors KF8 value at the absolute site 8 of the mutant peptide
BLOSUM62 derived indices BLOSUM10 value of site 1 and 2 compose-dipeptide of the mutant peptide
BLOSUM62 derived indices BLOSUM9 value of site 4 to 6 compose-tripeptide of the mutant peptide
Kidera factors KF10 value of site 7 to 9 compose-tripeptide of the mutant peptide
ST-scales ST4 value of site 2 and 3 compose-dipeptide of the mutant peptide
Cruciani properties PP1 value of site 3 and 4 compose-dipeptide of the mutant peptide
Kidera factors KF2 value of site 3 and 4 compose-dipeptide of the mutant peptide
Z-scales Z2 value of site 3 and 4 compose-dipeptide of the mutant peptide
FASGAI vector F4 value of site 3 and 4 compose-dipeptide of the mutant peptide
T-scales T1 value of site 3 and 4 compose-dipeptide of the mutant peptide
T-scales T4 value of site 3 and 4 compose-dipeptide of the mutant peptide
VHSE-scales VHSE2 value of site 3 and 4 compose-dipeptide of the mutant peptide
VHSE-scales VHSE3 value of site 3 and 4 compose-dipeptide of the mutant peptide
VHSE-scales VHSE6 value of site 3 and 4 compose-dipeptide of the mutant peptide
protFP descriptors protFP2 value of site 3 and 4 compose-dipeptide of the mutant peptide
protFP descriptors protFP4 value of site 3 and 4 compose-dipeptide of the mutant peptide
ST-scales ST1 value of site 3 and 4 compose-dipeptide of the mutant peptide
ST-scales ST4 value of site 3 and 4 compose-dipeptide of the mutant peptide
ST-scales ST5 value of site 3 and 4 compose-dipeptide of the mutant peptide
BLOSUM62 derived indices BLOSUM2 value of site 3 and 4 compose-dipeptide of the mutant peptide
BLOSUM62 derived indices BLOSUM7 value of site 3 and 4 compose-dipeptide of the mutant peptide
MS-WHIM scores MSWHIM1 value of site 3 and 4 compose-dipeptide of the mutant peptide
Cruciani properties PP1 value of site 4 and 5 compose-dipeptide of the mutant peptide
ST-scales ST3 value of site 4 and 5 compose-dipeptide of the mutant peptide
ST-scales ST5 value of site 4 and 5 compose-dipeptide of the mutant peptide
BLOSUM62 derived indices BLOSUM7 value of site 4 and 5 compose-dipeptide of the mutant peptide
BLOSUM62 derived indices BLOSUM9 value of site 4 and 5 compose-dipeptide of the mutant peptide
BLOSUM62 derived indices BLOSUM9 value of site 5 and 6 compose-dipeptide of the mutant peptide
Cruciani properties PP1 value of site 6 and 7 compose-dipeptide of the mutant peptide
Kidera factors KF10 value of site 6 and 7 compose-dipeptide of the mutant peptide
T-scales T4 value of site 6 and 7 compose-dipeptide of the mutant peptide
VHSE-scales VHSE6 value of site 6 and 7 compose-dipeptide of the mutant peptide
protFP descriptors protFP5 value of site 6 and 7 compose-dipeptide of the mutant peptide
BLOSUM62 derived indices BLOSUM8 value of site 6 and 7 compose-dipeptide of the mutant peptide

Cruciani properties PP3 value of site 7 and 8 compose-dipeptide of the mutant peptide
Kidera factors KF10 value of site 7 and 8 compose-dipeptide of the mutant peptide
Z-scales Z4 value of site 7 and 8 compose-dipeptide of the mutant peptide
FASGAI vector F6 value of site 7 and 8 compose-dipeptide of the mutant peptide
protFP descriptors protFP5 value of site 7 and 8 compose-dipeptide of the mutant peptide
ST-scales ST2 value of site 7 and 8 compose-dipeptide of the mutant peptide
Cruciani properties PP1 value of site 2 to 4 compose-tripeptide of the mutant peptide
Kidera factors KF2 value of site 2 to 4 compose-tripeptide of the mutant peptide
Z-scales Z2 value of site 2 to 4 compose-tripeptide of the mutant peptide
T-scales T1 value of site 2 to 4 compose-tripeptide of the mutant peptide
T-scales T4 value of site 2 to 4 compose-tripeptide of the mutant peptide
VHSE-scales VHSE2 value of site 2 to 4 compose-tripeptide of the mutant peptide
VHSE-scales VHSE6 value of site 2 to 4 compose-tripeptide of the mutant peptide
protFP descriptors protFP2 value of site 2 to 4 compose-tripeptide of the mutant peptide
ST-scales ST1 value of site 2 to 4 compose-tripeptide of the mutant peptide
ST-scales ST4 value of site 2 to 4 compose-tripeptide of the mutant peptide
ST-scales ST5 value of site 2 to 4 compose-tripeptide of the mutant peptide
BLOSUM62 derived indices BLOSUM2 value of site 2 to 4 compose-tripeptide of the mutant peptide
BLOSUM62 derived indices BLOSUM7 value of site 2 to 4 compose-tripeptide of the mutant peptide
MS-WHIM scores MSWHIM1 value of site 2 to 4 compose-tripeptide of the mutant peptide
Cruciani properties PP1 value of site 3 to 5 compose-tripeptide of the mutant peptide
T-scales T3 value of site 3 to 5 compose-tripeptide of the mutant peptide
VHSE-scales VHSE2 value of site 3 to 5 compose-tripeptide of the mutant peptide
VHSE-scales VHSE5 value of site 3 to 5 compose-tripeptide of the mutant peptide
protFP descriptors protFP4 value of site 3 to 5 compose-tripeptide of the mutant peptide
ST-scales ST4 value of site 3 to 5 compose-tripeptide of the mutant peptide
ST-scales ST5 value of site 3 to 5 compose-tripeptide of the mutant peptide
BLOSUM62 derived indices BLOSUM7 value of site 3 to 5 compose-tripeptide of the mutant peptide
BLOSUM62 derived indices BLOSUM9 value of site 3 to 5 compose-tripeptide of the mutant peptide
FASGAI vector F4 value of site 5 to 7 compose-tripeptide of the mutant peptide
T-scales T4 value of site 5 to 7 compose-tripeptide of the mutant peptide
VHSE-scales VHSE6 value of site 5 to 7 compose-tripeptide of the mutant peptide
protFP descriptors protFP5 value of site 5 to 7 compose-tripeptide of the mutant peptide
BLOSUM62 derived indices BLOSUM2 value of site 5 to 7 compose-tripeptide of the mutant peptide
BLOSUM62 derived indices BLOSUM9 value of site 5 to 7 compose-tripeptide of the mutant peptide
MS-WHIM scores MSWHIM1 value of site 5 to 7 compose-tripeptide of the mutant peptide
Kidera factors KF10 value of site 6 to 8 compose-tripeptide of the mutant peptide
Z-scales Z5 value of site 6 to 8 compose-tripeptide of the mutant peptide
VHSE-scales VHSE6 value of site 6 to 8 compose-tripeptide of the mutant peptide
protFP descriptors protFP5 value of site 6 to 8 compose-tripeptide of the mutant peptide
